# New Prediction Model for Probe Specificity in an Allele-Specific Extension Reaction for Haplotype-Specific Extraction (HSE) of Y Chromosome Mixtures

**DOI:** 10.1371/journal.pone.0045955

**Published:** 2012-09-25

**Authors:** Jessica Rothe, Norman E. Watkins, Marion Nagy

**Affiliations:** 1 Department of Forensic Genetics, Institute of Legal Medicine and Forensic Sciences, Charité- Campus Virchow-Klinikum, Berlin, Germany; 2 DNA Software, Inc., Ann Arbor, Michigan, United States of America; University of Louisville, United States of America

## Abstract

Allele-specific extension reactions (ASERs) use 3′ terminus-specific primers for the selective extension of completely annealed matches by polymerase. The ability of the polymerase to extend non-specific 3′ terminal mismatches leads to a failure of the reaction, a process that is only partly understood and predictable, and often requires time-consuming assay design. In our studies we investigated haplotype-specific extraction (HSE) for the separation of male DNA mixtures. HSE is an ASER and provides the ability to distinguish between diploid chromosomes from one or more individuals. Here, we show that the success of HSE and allele-specific extension depend strongly on the concentration difference between complete match and 3′ terminal mismatch. Using the oligonucleotide-modeling platform *Visual Omp,* we demonstrated the dependency of the discrimination power of the polymerase on match- and mismatch-target hybridization between different probe lengths. Therefore, the probe specificity in HSE could be predicted by performing a relative comparison of different probe designs with their simulated differences between the duplex concentration of target-probe match and mismatches. We tested this new model for probe design in more than 300 HSE reactions with 137 different probes and obtained an accordance of 88%.

## Introduction

**Figure 1:**
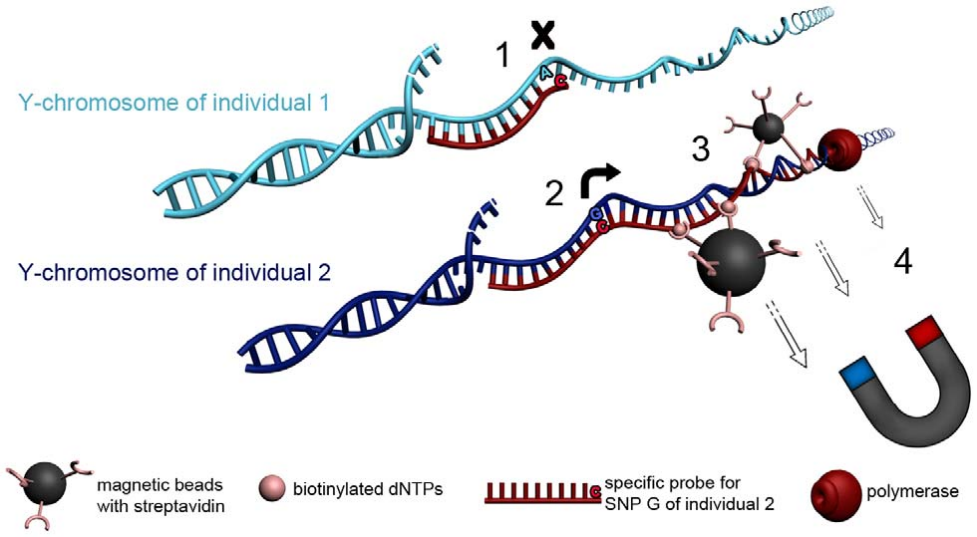
Schematic illustration of HSE for the separation of male DNA mixtures. A male DNA mixture is shown by the presents of two different Y-chromosomes from individual 1 (light-blue) and individual 2 (dark-blue) which differ in a theoretical SNP A/G. The principal of HSE is than shown by the separation of a Y-chromosomal fragment of individual 2 by the use of a probe specific to the SNP allele G. (1) HSE-probe shows 3′terminal mismatch for individual 1, therefore no extension occurs. (2) Probe matches completely at Y-chromosome of individual 2 and can be extended. (3) During extension reaction the polymerase incorporates biotinylated dNTPs. Streptavidin coated magnetic beads bind to biotin labeled DNA. (4) The DNA-biotin-streptavidin complex can be captured by the use of a magnetic field.

Allele-specific extension reactions (ASERs) are used for a broad range of applications, especially in the detection of single nucleotide polymorphisms (SNPs) for medical usage, disease association studies, and routine molecular diagnostics [1]–[3]. One very elegant application of ASERs is haplotype-specific extraction (HSE), which is used routinely for the resolution of ambiguous detected human leukocyte genotypes in respect to clinical applications and transplantation [4]–[7]. Thermostable Taq DNA polymerase is used in HSE for the extension of an allele-specific probe, followed by 20 minutes of elongation with the incorporation of biotinylated nucleotides and an extraction step with streptadivin-coated magnetic beads (Figure 1). Therefore, HSE allows the separation of coherent chromosomal fragments or haplotypes, as well as numerous applications for different scientific approaches [8]–[10]. The present study demonstrates the successful application of the HSE protocol for the separation of male DNA mixtures. In forensic work DNA mixtures arise when two or more individuals contribute to the same sample, which can occur in a number of evidentiary situations, such as fingernail clippings or swabs taken from the skin or body orifices. HSE allows the separation of different contributors from a DNA mixture and therefore avoid complicated interpretations of mixed DNA profiles. For all applications of ASER, the reliability and specificity of the approach is dependent on the ability of the DNA polymerase to discriminate between the extension of mispaired and canonically paired primers. Because polymerase binds with similar affinities to matched and mismatched primer templates, the non-specific extension of a 3′ nucleotide mismatch is often a significant fraction of the overall extension product, which can be controlled with rational probe design [11].

Adaptation of HSE to forensics for the purpose of separating male DNA mixtures has already been presented in the literature in regards to the influence of the buffer composition (dNTPs, biotinylated nucleotides, and Taq polymerase) on template-specific polymerase activity [12]. However, to obtain optimum separation efficiency in HSE, the the probe design must be addressed. For this purpose, the ability of primer 3′ terminal nucleotides to discriminate SNPs in templates was qualitatively analyzed in regards to base identity and mismatch discrimination. However, a more quantitative approach to HSE allele-specific probe design would result in more efficient probes and successful separation of male DNA mixtures. Therefore, the thermodynamic considerations of optimal allele-specific probe design must be considered [13]–[14].

Ayyadevare *et al.* [15] reported that template discrimination was optimal when the nucleotides T, G, or C, but not A, occupied the 3′ position of a primer. This group went on to investigate the effect that nucleotide identity has on template discrimination from the penultimate (−1, or nearest neighbor) position relative to the 3′ end of the primer and reported that amplification efficiency was reduced when T or A occupied this position. The thermodynamic effects of primer and probe nucleotides and the effects that their “nearest neighbors” have on the stability of base-pairing were quantified by SantaLucia *et al.* [16], who determined the enthalpic and entropic contributions of nearest neighbor match and mismatch sequence composition, base stacking, and mismatch geometry [17]–[20]. These nearest neighbor parameters are available in the oligonucleotide design and simulation software platform *Visual OMP* (DNA Software, Inc., Ann Arbor, MI, USA). *Visual OMP* can design allele-specific HSE primers within user-defined design criteria and experimental conditions and simulate probe designs to check for specific duplex hybridization, and also for competing equilibria that may result in the unsuccessful separation of male DNA mixtures.

In the present study, data from 300 HSE reactions are combined with data from 500 simulations in *Visual OMP* to correlate the influence of HSE probe length with assay efficiency by testing the separation of a male DNA mixture, using six different forensic short tandem repeat (STR) markers, at nine different extraction loci.

## Materials and Methods

### Selection of DNA Samples and HSE of DNA from Male DNA Mixtures

The four human samples in use are male DNA reference samples of the laboratory. The laboratory members provided their informed written consent to project investigators in line with the Declaration of Helsinki. The current study was approved by the institutional review board of the Institute of Legal medicine Berlin under protocol authorization number 01_08_2008_01. DNA mixtures were separated using the EZ1 HaploPrep Kit (Qiagen) and a protocol designed by Dapprich *et al.* [21]. All extractions were set up in 30 µl reactions containing 300–500 ng genomic DNA, 5 mM of probe (TIP MOLBIOL Berlin, Germany), and 1X hybridization buffer (HB), which contained biotinylated dNTPs in addition to standard dNTPs, polymerase, MgCl_2_, and DNAse-free water. Individual DNA samples were denatured at 95°C for 7.5 minutes on an external heating block with a heated lid (True Temp; Robbins Scientific Corp., Sunnydale, CA, USA). The samples were then transferred and incubated at 64°C for 20 minutes in a BioRobot® EZ1 Workstation to produce amplicons from the DNA samples that contained biotinylated dNTPs. The biotinylated amplicons were captured using streptavidin-coated magnetic microparticles, washed twice with washing buffer, and re-suspended in 50 µl of elution buffer. Successful isolation of the male DNA Y-chromosome fragments for HSE was achieved using an AmpFℓSTR® Y-filer kit (Applied Biosystems, New Jersey, USA). The magnetic beads were removed from the biotinylated amplicons by incubating the samples at 75°C for 10 minutes, followed by AmpFℓSTR® Yfiler multiplex PCR per the manufacturer’s instructions [12].

### Selection of SNPs for HSE Probes

The selection of SNPs for HSE probe design was based on two criteria: the distance of the SNP from the nearest Y-STR system included in the AmpFℓSTR® Yfiler kit, and the variability of the SNP within the European population. Therefore, the SNPs P30, P38, P224, P240, P244, Tat, and rs13304202 were selected from the dbSNP (Single Nucleotide Polymorphism database NCBI; http://www.ncbi.nlm.nih.gov/snp/) and Family Tree databases (http://ymap.ftdna.com). The existence of the SNPs in the DNA samples was confirmed by a pyrosequencing protocol using a PSQ 96 MA robot (Biotage, Uppsala, Sweden) according to the manufacturer’s instructions (File S1A, S1C). In addition to the SNPs selected from the NCBI database, two additional SNPs discovered by Sanger sequencing of a 4 Kb fragment of the DYS437 flanking region in the obtained samples (Data S1B), SeqE2071 and SeqF4204, were included in the study.

### Probe Design for HSE

**Figure 2:**
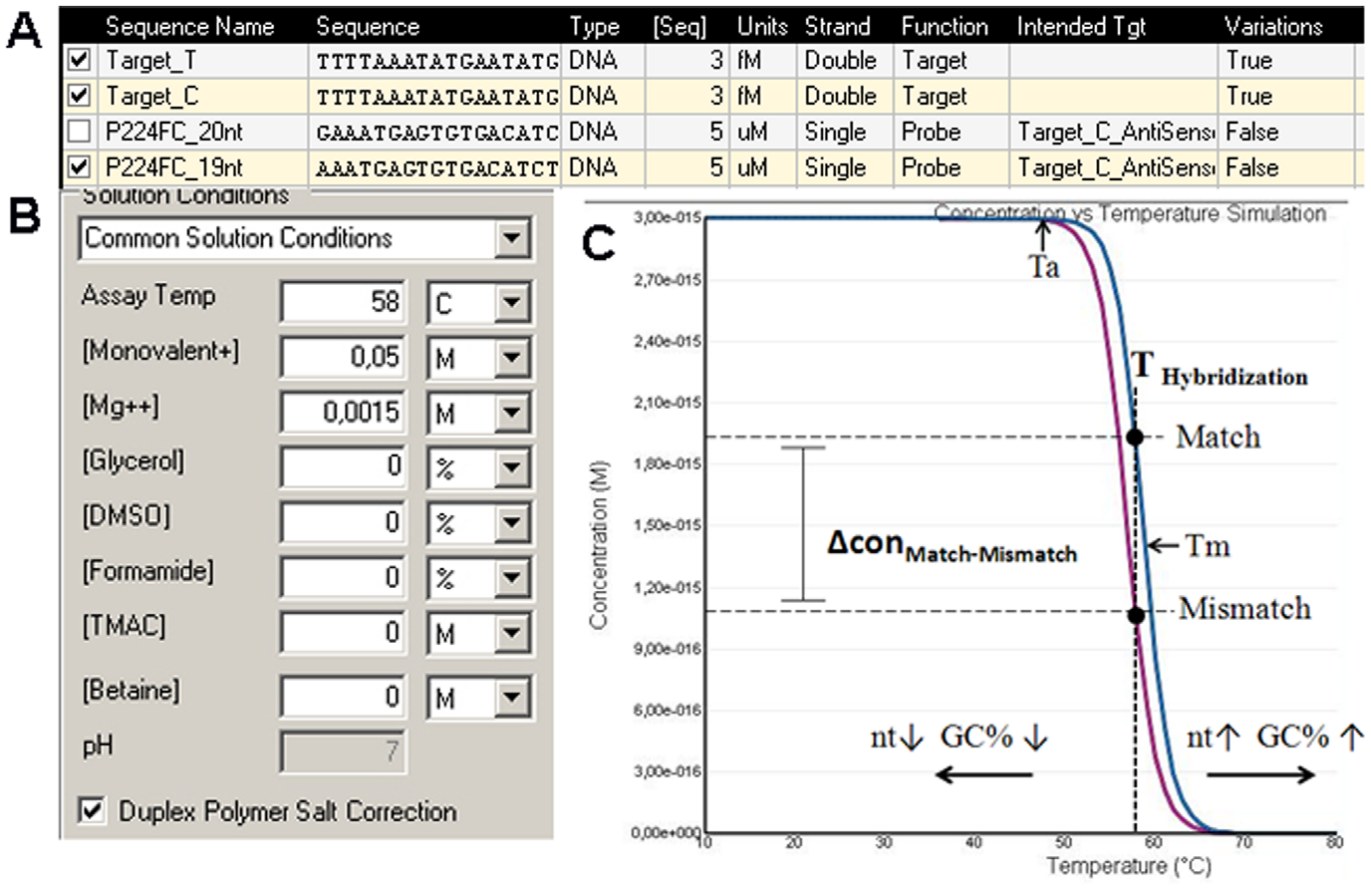
Direct comparison of the thermodynamic stability of match and mismatch using *Visual OMP*. The program allows the simulation of the concentration of hybridized matches and mismatches by adding the starting concentration (A) of the target and probe, as well as assay parameter (B) under the solution conditions. (C) Simulation of concentration versus temperature. Schematic representation of the comparison between match and mismatch by concentrations. Ta, optimal annealing temperature for PCR; Tm, melting temperature. Horizontal arrows indicate a switch in the hybridization curve for decreasing (↓) or increasing (↑) probe length (nt) and G/C content (GC%).

Allele-specific HSE probes were designed for each of the nine referenced SNPs, and each SNP (extraction loci) had the potential for eight different design variants; probes were designed either in forward or reverse orientation with the discriminating nucleotide at either the 3′ or penultimate (−1) position. Each probe variant was designed for different probe lengths, which together we refer to as one “probe set”. Different probe lengths in one probe set were then tested in HSE to identify the length with the best separation effect. Table 1 provides the probe nomenclature, mismatch type, nucleotide sequence, and sequence lengths used in this study. For the prediction of best probe length, melting curve analysis was performed for each specific probe or probe set in *Visual OMP,* which plots duplex concentration as a function of temperature (DNA Software, Inc.) [17], [18]. Thus, the change in duplex concentration for match and mismatch probes (Δcon_M-MM_) for a specific allele could be determined, as shown in Figure 2.

**Table 1:** Probes tested in HSE.

probe^1^	mismatch	sequence	tested different probe-length in nucleotides^2^
P30FG	G-T	CAGGTGATAGATAAGTTGATCG	17, 19–22
P30FG -1	G-T	GGTGATAGATAAGTTGATCGA	21
P30FA	A-C	CAGGTGATAGATAAGTTGATCA	17, 19, 22
P30FA -1	A-C	AGGTGATAGATAAGTTGATCAA	21, 22
P30RC	C-A	TCTATCCATCTATCATCTATTTATC	19, 21, 24, 25
P30RC -1	C-A	TCTATCCATCTATCATCTATTTATCG	23, 26
P38FA	A-G	GCTGGGAGGGTGGCTCCCGCA	10–13, 15, 16, 19–21
P38FA -1	A-G	GGGAGGGTGGCTCCCGCAT	12, 16, 19
P38FC	C-T	CTGGGAGGGTGGCTCCCGCC	10, 12, 13, 15, 18–20
P38FC -1	C-T	GGAGGGTGGCTCCCGCCT	12, 18
P224FC	C-A	TCAGAAATGAGTGTGACATCTTC	9–21, 23
P224FT	T-G	TCAGAAATGAGTGTGACATCTTT	18–21, 23
P224RG	G-T	GTGGTTTCAGTCAGCAGGGG	17, 20
P224RA	A-C	GTGGTTTCAGTCAGCAGGGA	17, 20
P240FC	C-A	TCTTTCAGATCAATAACGTCTC	17, 19–22
P240FC -1	C-A	TTTCAGATCAATAACGTCTCG	18–21
P240FT	C-A	TCTTTCAGATCAATAACGTCTT	22
P240FT -1	C-A	TTTCAGATCAATAACGTCTTG	20, 21
P240RG	G-T	GTAGGCTCAGATAAAGAACG	16–20
P240RG -1	G-T	TAGGCTCAGATAAAGAACGA	19, 20
P240RA	A-C	GGTAGGCTCAGATAAAGAACA	17–21
P244FA	A-C	CAGTGCAACAGGACCA	14, 16, 23
P244FA -1	A-C	AGTGCAACAGGACCAG	15, 16
P244FG	G-T	GCCCAGCAGTGCAACAGGACCG	11–16, 23
P244FG -1	G-T	GTGCAACAGGACCGG	13–15
P244RC	C-A	TATTGTCCTGCAGCTCCATCCCC	13–15, 23
P244RC -1	C-A	CAGCTCCATCCCCG	13, 14
P244RT	T-G	ATTGTCCTGCAGCTCCATCCCT	11, 13, 16, 22
P244RT -1	T-G	CAGCTCCATCCCTG	14
TatFT	T-G	GTGTAGACTTGTGAATTCAT	20
TatFC	C-A	GTGTAGACTTGTGAATTCAC	18, 20
rs13304202FA	A-C	TAAGGAACATTACTCAAGAGA	18–21
rs13304202FA -1	A-C	TAAGGAACATTACTCAAGAGAC	20, 22
rs13304202FG	G-T	TAAGGAACATTACTCAAGAGG	21
rs13304202FG -1	G-T	GGAACATTACTCAAGAGGC	19
rs13304202RC	A-C	AGTTTTATTTATGGAGGAAGC	18, 19, 21
rs13304202RC -1	A-C	TTAGTTTTATTTATGGAGGAAGCC	19–21, 24
Seq.E2071 RG	G-T	AGGCTGTGCTATTGATGAAAATG	20, 23
Seq.E2071 RA	A-C	AGGCTGTGCTATTGATGAAAATA	20, 23
Seq.F4204 FG	G-G	GGTCTTCCTCTGTTCCTCAG	17, 20
Seq.F4204 FC	C-C	GGTCTTCCTCTGTTCCTCAC	17, 20

1 Probes were named after the corresponding SNP, orientation (F  =  forward, R  =  reverse), and discriminating 3′ terminal base, which is also underlined in the sequence. Probe names have been denoted with −1 when the discriminating base is placed on the second base-pair of the 3′ terminus.

2 Numbers refer to the different lengths of the probes that were tested. For example, probe rs13304202FA was tested using lengths of 18, 19, 20, and 21 nucleotides, whereas the probes have been shortened step-wise on their 5′ termini.

### HSE Probe Efficiency

The efficiency of allele-specific HSE probes was evaluated using Equation 1. The HSE probe efficiency was then categorized as complete separation at >89%, significant separation at ≥61%, and no separation at ≤60% according to the presence of short tandem repeat (STR) markers in the amplicons. The STR markers were DYS389I, DYS389II, DYS390, DYS437, DYS439, and DYS635. Standard deviations were calculated for each STR marker from the Y-filer analysis of unseparated mixtures.
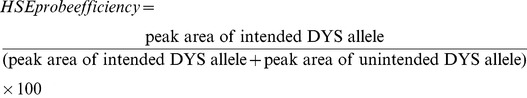
(1)


The observed standard deviations for each STR per number of controls (n) were: DYS389I, DYS389II: 8.8% (n = 25), DYS390∶7.9% (n = 88), DYS437∶7.0% (n = 94), DYS439; 6.8% (n = 75), DYS635 8.0% (n = 95), and DYS438∶8.8% (n = 95).

Probe efficiency could be influenced by additional hybridization of the probe near the extraction locus. To assess probe specificity the probe sequence was aligned to 50 kb sequence, up- and downstream of the extraction locus with the program FastPCR 6.0. The limit of 50 kb correlates with the maximum distance between extraction locus and STR marker at which successful separation by HSE has been detected (data not shown).

## Results

### Influence of Probe Length on HSE Specificity Using P224 as an Example

**Figure 3:**
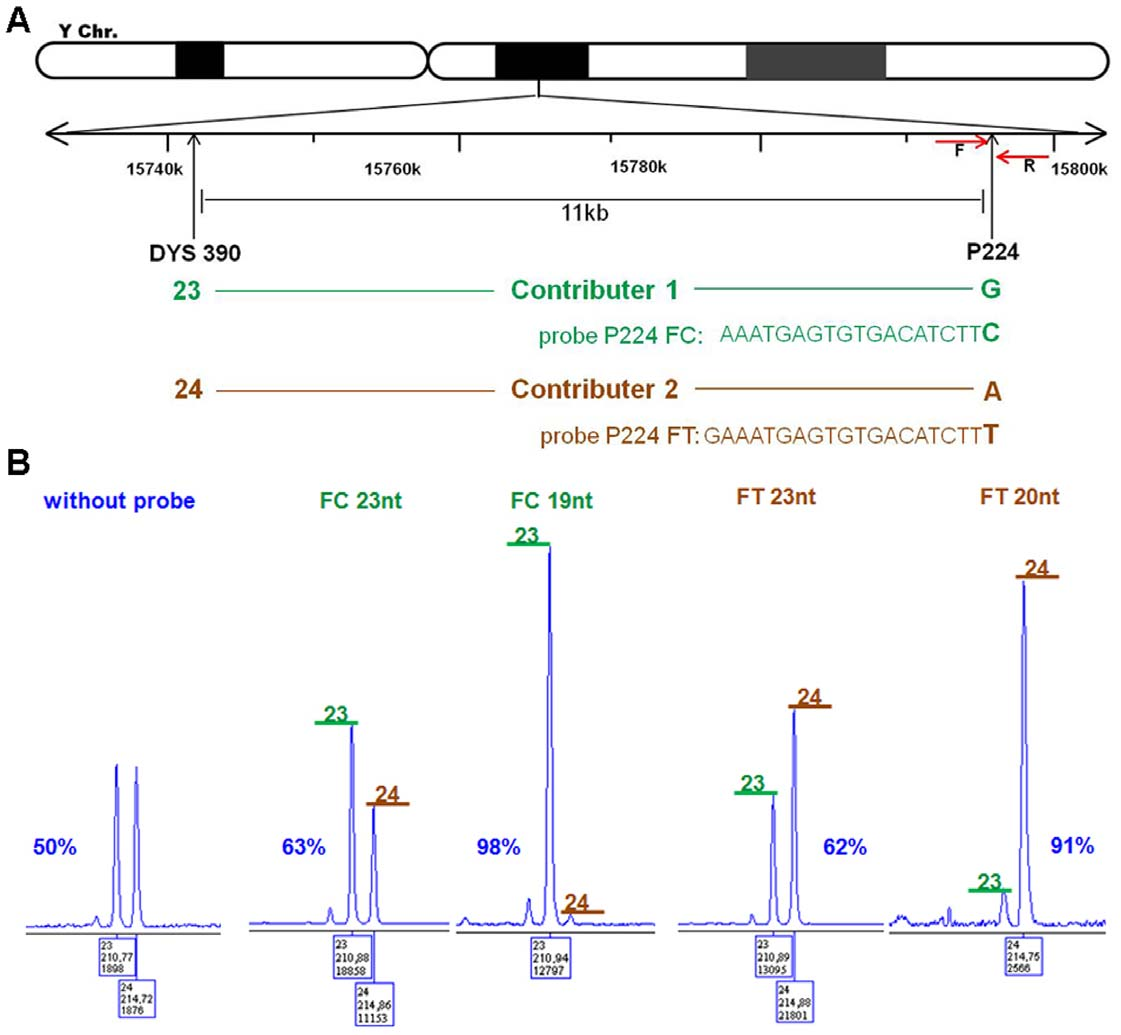
Specific extraction at the P224 locus by HSE and evaluation by AmpFℓSTR® Yfiler PCR. (A) Map of the 11 kb distant DYS390 and P224 loci on the human Y chromosome, which were selected for HSE analysis. Arrows indicate the positions of the probes used (RG, FC, FT, or RA), which were orientated in either the forward (F) or reverse (R) direction. Given chromosome positions are from a reference sequence (Hg18). (B) Electropherograms of AmpFℓSTR® Yfiler analysis of haplo-separated samples obtained from a DNA mixture (Contributor 1 and Contributor 2) using the probes P224FC (23 and 19 nucleotides long) and P224FT (23 and 20 nucleotides long), or without probes. Green bars with allele 23 correspond to Contributor 1, and brown bars with allele 24 correspond to Contributor 2. Percentages indicate the enrichment of one Contributor.

**Figure 4:**
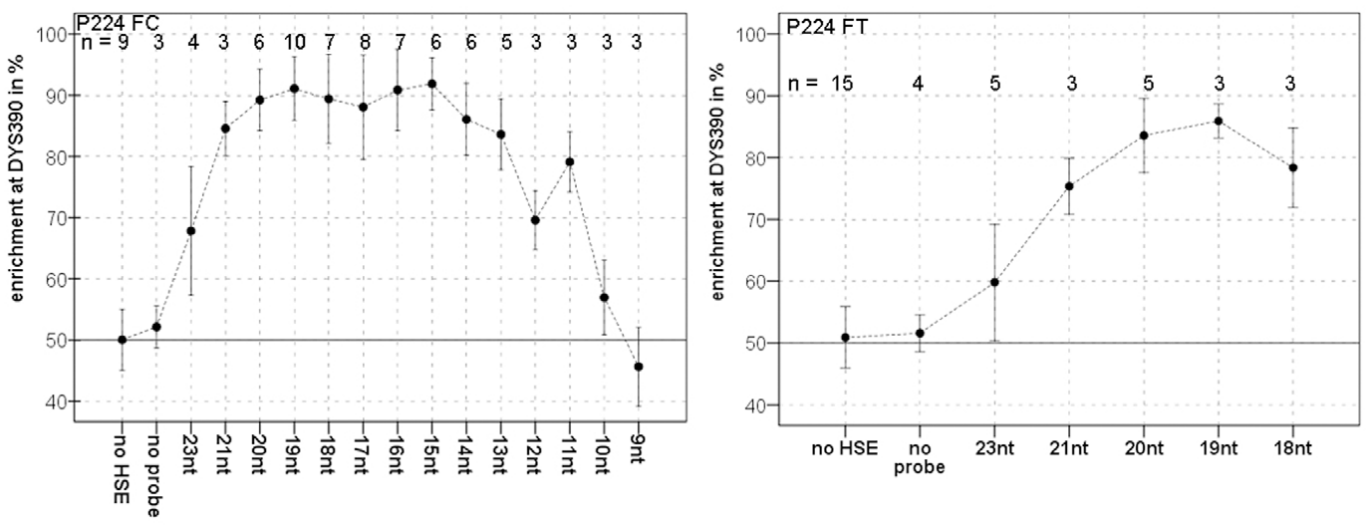
Separation by HSE with different probes. Enrichment of one contributor from a male DNA mixture is shown in relation to probe length for two different probe sets P224FC/FT (see also File S2). The bars indicate standard deviation of the mean enrichment. No HSE indicates analysis of the male DNA mixture without separation by HSE, and no probe indicates separation of a male DNA mixture by HSE without probe. n  =  number of extractions.

For the separation of male DNA mixtures in a forensic application of HSE, allele-specific probes were designed adjacent to informative STR markers on the Y chromosome (included in the Y-filer) to allow allele-specific extension by the Taq polymerase. Therefore, probe design is limited by the specified extraction locus and hybridization temperature, which restricts design options to orientation and length. Figure 3A demonstrates probe design using probe P224, which was designed for the phylogenetic SNP P224 11 kb from the marker DYS390, as an example. The extraction site SNP P224 was used first in studies of probes with two different lengths for each orientation. HSE was carried out with one probe and a male DNA mixture containing two contributors that differ at both loci, DYS390 and P224. As in forensic analysis, Y-filer was used to detect the DYS390 locus of the HSE-P224 samples, which reflects the separation effect of the DNA mixture. The proportions obtained for the two alleles (DYS390 23 and 24) indicate the success of the separation of the male DNA mixture by HSE. The first probe design studies showed that HSE without a probe results in no enrichment of one contributor allele, whereas the use of probes increases the presence of one contributor allele. The 23-nucleotide probes, led to an approximately 60% enrichment of the corresponding contributor (Figure 3B). Surprisingly, probes that were only three or four nucleotides shorter exhibited a strong increase in the separation of one contributor to nearly 100%. The orientation of the probe did not have any significant effect. A more comprehensive study of probe P224FC comprising tests of decreasing probe length (21 and 23 nucleotides to 9 nucleotides) revealed optimum HSE success with a length between 15 and 20 nucleotides, whereas longer or shorter probes led to a rapid decrease in HSE success. In addition, the probe set P224FT was found to a similar curve with an optimum of 19 nucleotides (Figure 4).

### Prediction of Probe Specificity

To understand the dependence of HSE on probe length, we tested 41 probe sets for the nine extraction loci: P30, P38, P240, P244, Tat, rs13304202, Seq.E2071, and Seq.F4204. In total we tested 137 different HSE probes in 345 HSEs. Table 2A provides a summary of all tested probe sets with the best probe lengths for highest HSE success, corresponding G/C content, distance to the closed STR marker, and the BLAST results of each probe. Our results confirmed the presence of an optimum probe length for HSE, but showed that the size and range of the optimum length vary greatly for each extraction locus. The variation in the maximum enrichment of one contributor is generally caused by the different distance of each extraction locus and sequence specificity of the probe.

**Table 2:** Summary of all analyzed probes in this study.

A						B	C		D	
probe (distance to STR marker)		mean GC%	distance of hits in kb with matching 3′end 1	HSE results 3		ΔΔG _(M-MM)_	simulation with Viusal Omp		Match (Target-Probe)	Mismatch (Target-Probe)
				success in %	best tested length		best length	HSE fits with simulation		
**P30 (18 kb)**	**FG**	36	no	**76**	21*	−1,15	21, 22	**r**	C-G	T-G
	**FG-1**	35	no	**82**	21	−1,80	21	**r**	C-G	T-G
	**FA**	33	no	**83**	20*	−0,40	21	**r**	T-A	C-A
	**FA-1**	30	no	**95**	22	−1,01	22	**r**	T-A	C-A
	**RC**	27	no	**92**	24*	−0,49	24	**r**	G-C	A-C
	**RC-1**	30	2, 24	**70**	23	−2,18	23, 27	**r**	G-C	A-C
**P38 (17 kb)**	**FA**	77	no^2^	**89**	21	−0,60	11	**w**	T-A	A-G
	**FA-1**	71	no^2^	**86**	20	−1,04	12	**w**	T-A	A-G
	**FC**	83	2, 9, 10, 13+	**72**	15	−1,19	10	**w**	G-C	T-C
	**FC-1**	79	7, 10+	**49**	n.s.	−1,83	12	**w**	G-C	T-C
**P224 (13 kb)**	**FC**	40	no	**90**	19*	−0,37	18	**r**	G-C	A-C
	**FT**	34	no	**86**	19*, 20*	−0,70	19	**r**	A-T	G-T
	**RG**	60	no	**93**	17	−1,31	14	**r**	C-G	T-G
	**RA**	53	no	**86**	17	−0,48	15, 16	**r**	T-A	C-A
**P240 (14 kb)**	**FC**	37	no	**82**	22*	−0,48	22	**r**	G-C	A-C
	**FC-1**	40	no	**83**	20*	−2,43	20, 21	**r**	G-C	A-C
	**FT**	32	no	**84**	22	−0,60	22	**r**	A-T	T-C
	**FT-1**	35	no	**94**	20	−1,52	20, 21	**r**	A-T	T-C
	**RG**	42	no	**79**	18*	−1,14	19	**r**	C-G	T-G
	**RG-1**	40	no	**73**	20	−1,21	20	**r**	C-G	T-G
	**RA**	39	no	**88**	18	−0,87	18, 20	**r**	T-A	C-A
**P244 (35 kb)**	**FG**	66	49	**73**	13*	−0,95	15	**r**	C-G	T-G
	**FG-1**	65	no	**78**	15	−2,11	14	**r**	C-G	T-G
	**FA**	57	116+	**54**	n.s.	0,05	no	**r**	T-A	C-A
	**FA-1**	59	0,6+	**76**	15	−1,04	15	**r**	T-A	C-A
	**RC**	68	20, 29+	**68**	14*	−0,66	13	**r**	G-C	A-C
	**RC-1**	71	20+	**71**	14	−2,16	14	**r**	G-C	A-C
	**RT**	60	2, 9, 13+	**62**	13	−0,69	13	**r**	A-T	G-T
	**RT-1**	64	9, 13+	**66**	14	−1,01	14	**r**	A-T	G-T
**Tat (15 kb)**	**FT**	33	no	**84**	20	−0,57	20	**r**	T-A	G-T
	**FC**	38	no	**87**	18	−0,52	20	**r**	G-C	A-C
	**FG**	40	no	**75**	19	−1,39	20	**r**	C-G	T-G
	**FG-1**	43	no	**81**	18	−2,77	19, 20	**r**	C-G	T-G
**rs13304202 (37 kb)**	**FA**	34	no	**77**	19	0,42	no	**w**	T-A	C-A
	**FA-1**	38	no	**76**	22	−1,55	20, 22	**r**	T-A	C-A
	**RC**	39	no	**70**	21	−1,17	22	**r**	G-C	A-C
	**RC-1**	43	no	**81**	21*	−3,00	21, 24	**r**	G-C	A-C
**Seq.E 2071 (0.3 kb)**	**RG**	35	no	**78**	20	−1,19	19	**r**	C-G	T-G
	**RA**	30	no	**91**	20	−0,13	20	**r**	T-A	C-A
**Seq.F 4204 (0,15 kb)**	**FG**	53	0.3 kb with matched 3′ end	**67**	17	−0,71	18	**r**	C-G	G-G
	**FC**	53	0.3 kb but no matched 3′ end	**98**	17	−0,83	14	**r**	C-G	C-C

**(A)** and **(B)** Comparison of HSE results for all probe sets with distance to the STR marker, G/C content, hit frequency, and free energy differences of match and mismatch in kcal/mol.

1 The number is the distance in kb of strong hits to the extraction locus, + indicates the presence of further hits but greater distance (>30 kb).

2 Best simulated probes P38FA (11 nucleotides long) and P38FA-1 (12 nucleotides long) show many hits (4, 12, 16 kb+ and 4, 10 16 kb+).

3 HSE results show the best experimentally evaluated probe length with enrichment of one contributor. Low separation in HSE could be due to a higher hit frequency close to the extraction locus and great distance from the STR marker. Asterix indicate the best probe length. **(C)** The best probe length was evaluated after simulation and chosen after highest Δcon, and comparison of the prediction for best probe length after Δcon and best probe in HSE: (r) right prediction, (w) wrong prediction. **(D)** Listing of all analyzed match and mismatch constellations in this study. List of complete data is available in Data S3.

For an initial summary of the results, we compared the separation success in HSE for all investigated probes to probe length, free energy, melting temperature, and G/C content. Table 3A provides these results for the P224FC and P30RC probe set. As probe length increased, melting temperature and free energy also increased, though probe G/C content is a function of sequence identity. Optimum separation was obtained using probes 15–20 nucleotides in length that ranged in melting temperature from 54–59°C, free energy of −6.56 to −8.69 kcal/mol, and G/C content of 40 to 47%. Comparatively, for the P30RC probe set, optimum separation was obtained with probes 24 or 25 nucleotides in length with a melting temperature of 57°C, and free energy of −7.63 to −7.55 kcal/mol, and G/C content of 28 to 29%. This data demonstrates that the differences in target sequence complexity from one target to the next must determine the optimum design attributes for each probe, whereas these attributes are not reliable indicators of HSE efficiency.

**Table 3:** Probe design for P224 FC and P30RC.

A								B		
probe (STR marker)	l	probe sequence	GC%	Tm_M_	ΔG°_M_	HSE result in %	n	con_M_*10^−17^M	con_MM_*10^−17^M	Δcon_M-MM_*10^−17^M
										
**P224FC (DYS 390)**	25	TTTCAGAAATGAGTGTGACATCTTC	36	63	−10,97	–		297	290	**7**
	24	TTCAGAAATGAGTGTGACATCTTC	38	63	−11,09	–		297	292	**6**
	23	TCAGAAATGAGTGTGACATCTTC	39	63	−10,73	**68±11**	4	295	285	**10**
	22	CAGAAATGAGTGTGACATCTTC	41	63	−10,80	–		296	287	**9**
	21	AGAAATGAGTGTGACATCTTC	38	61	−9,58	**85±4**	3	274	233	**41**
	20	GAAATGAGTGTGACATCTTC	40	59	−8,69	**91±5**	6	219	141	**79**
	19	AAATGAGTGTGACATCTTC	37	59	−8,32	**89±5**	10	182	100	**82**
	18	AATGAGTGTGACATCTTC	39	58	−7,95	**91±7**	7	141	68	**73**
	17	ATGAGTGTGACATCTTC	41	57	−7,58	**88±9**	8	100	43	**58**
	16	TGAGTGTGACATCTTC	44	54	−6,40	**91±7**	7	23	9	**14**
	15	GAGTGTGACATCTTC	47	54	−6,56	**92±4**	6	29	12	**17**
	14	AGTGTGACATCTTC	43	52	−6,02	**86±6**	6	13	5	**8**
	13	GTGTGACATCTTC	46	49	−5,15	**84±6**	5	4	1	**2**
	12	TGTGACATCTTC	42	45	−4,26	**70±5**	3	1	0	**1**
	11	GTGACATCTTC	45	44	−4,01	**79±5**	3	1	0	**0**
	10	TGACATCTTC	40	36	−2,85	**57±6**	3	0	0	**0**
	9	GACATCTTC	44	35	−2,60	**54±7**	3	0	0	**0**
	8	ACATCTTC	38	26	−2,06	–		0	0	**0**
	6	ATCTTC	43	21	−0,93	–		0	0	**0**
**P30RC (DYS 439)**	26	TTCTATCCATCTATCATCTATTTATC	27	**57**	−7,43			86	48	**38**
	25	TCTATCCATCTATCATCTATTTATC	28	**57**	−7,55	**86±1**	2	97	56	**42**
	24	CTATCCATCTATCATCTATTTATC	29	**57**	−7,63	**92±9**	2	106	61	**44**
	23	TATCCATCTATCATCTATTTATC	26	**54**	−6,02	–		13	7	**7**
	22	ATCCATCTATCATCTATTTATC	27	**55**	−6,62	**51±1**	2	31	16	**16**
	21	TCCATCTATCATCTATTTATC	28	**53**	−5,47	–		6	3	**3**
	20	CCATCTATCATCTATTTATC	30	**54**	−6,04	–		14	7	**7**
	19	CATCTATCATCTATTTATC	26	**49**	−4,14	**46±8**	2	1	0	**0**
	18	ATCTATCATCTATTTATC	22	**47**	−3,56			0	0	**0**

Probe P224FC and P30RC were designed for SNP P224 (C) and SNP P30 (G) as the extraction loci in forward (F) and reverse (R) orientation. (A) For different probe length (l), the G/C content, melting temperature (Tm), and ΔG value were calculated using *Visual Omp*. The success of separation by HSE with different probe lengths is given as the percent enrichment of one contributor with standard deviation and number of experiments (n). (−) indicates no data (B) Concentrations of hetero-duplex target probes (con) for match (M) and mismatch (MM) have been simulated for different probe lengths using Visual OMP with the assay parameters. Δ con presents the difference between match and mismatch concentrations.

Next, we compared match and mismatch hybridization by calculating ΔΔG_M-MM_ and simulating the concentration of match (M) and mismatch (MM) probes (Table 2B). Within one probe set, ΔΔG increased slowly with probe length but did not exhibit any coherency with probe-specificity in HSE (Figure S4B). Comparison of the ΔΔG values between different probe sets did not reveal a correlation between the extent of ΔΔG and HSE success or the type of mismatch. For example, the probes rs1330402RC and P240FC both had a G-C mismatch, but their ΔΔGs were −1.17 and −0.48 kcal/mol, respectively, which are relatively very different free energy contributions. The probes P224 FC and RG had very different ΔΔG values, −0.37 and −1.31 kcal/mol, respectively, but both had very high enrichment (>90%) of one contributor (Table 2B). The simulated match and mismatch concentrations increased with probe length, but their concentration difference (Δcon_ M-MM_) exhibited an optimum curve dependent on probe length. For example, the simulation for P224FC showed that, with increasing probe length, Δcon first increased and then decreased dramatically when probes were longer than 21 nucleotides (Table 3B). For all other tested probe sets, except P244FA and rs13304202FA, we observed a similar increase and decrease of Δcon with longer probes. The observation of an optimum Δcon curve reflects the separation effect of the tested probes fairly well. For example, the simulation of Δcon for probe P224 FC showed a broad optimum for probe length of 17 to 21 nucleotides and maximal HSE success with 13 to 21 nucleotides. For probe P224FT, optimum Δcon occurred only for a shorter range of 19 and 20 nucleotides, which completely agrees with the observed separation maximum for HSE (File S3). Thus, single Δcon values do not provide information about probe specificity but have to be regarded as relative values within one probe set to determine the best probe length for the maximal concentration difference between matched and mismatched probes. For example, the maximum Δcon between probe sets can have very different values but still exhibit the same probe specificity. For our nine selected extraction loci, we simulated over 500 probes and compared them to the separation success of 345 HSEs. In total, 88% (36 probe sets) of the analyzed data showed accordance of the obtained HSE results with the simulated Δcon curve (Table 2C). Therefore, we observed within one probe set that the probes with simulated maximum Δcon correlate with HSE success. However, five probe sets (12%), which refer to probe P38 and rs13304202 FA, did not show any accordance between Δcon and HSE success (Table 2C). The maximum simulated Δcon for probe P38 occurred with 11 or 12 nucleotides, but in HSE the optimum was at a longer probe length (<19 nucleotides). For rs13304202FA, *Visual OMP* predicted that mismatches were more stable than their matches. Therefore, no separation by HSE was predicted, though some separation was observed. In the case of P244FA, matched and mismatched targets were observed in which both free energies were predicted to be equal and, as expected, no specific extension was observed.

### Analysis of the Data According to Mismatch Position, Mismatch Type, and G/C Content of Probes

For probe sets with a low separation effect, we also tested probes with an internal mismatch at the −1 position of the 3′ termini. In total, we tested 15 internal mismatches and their associated probes with terminal mismatches (Table 2A). An increased enrichment of over 10% was observed for only three probe sets (rs13304202RC, P240FT, and P30FA) when the mismatch was located at the penultimate position. In contrast, probe sets P30RC-1 and P38FC-1 exhibited a decrease in enrichment of 22% and 23%, respectively. We tested nine different types of mismatches; the mismatches (target-probe) A-C, C-A, and T-G occurred with the highest frequency (Table 2D). A comparison of seven A-C/C-A mismatches contra G-T/T-G mismatches did not reveal any significant effect on HSE success for one mismatch. However, more comprehensive data are necessary for better evidence of the influence of type and position of mismatch for specific enzymatic extension reactions.

The target hybridization efficiency of six core probe match and mismatch sets was simulated in *Visual OMP*. Each of the core probe sets ranged in length from 6 to 46 base pairs varying in G/C content from 32% to 77% as length increased. Each probe length was plotted as an independent variable against the dependent variables of duplex free energy (ΔG) and probe concentration (con) (File S4A and C). A clear dependence was observed between probe length, G/C content, and concentration, as well as for ΔG, as expected, though no correlation with HSE efficiency was observed. Next, the changes in free energy (ΔΔG) and concentration (Δcon) were compared among the six core probe match and mismatch sets based on probe length. For the plot of ΔΔG as a function of probe length, no correlation with HSE efficiency was observed (Figure S4B).

**Figure 5:**
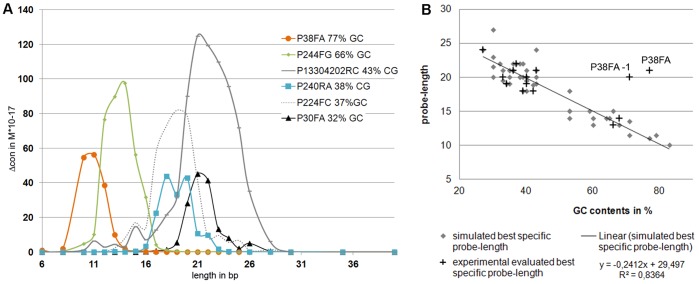
Influence of the G/C contents on Δcon and HSE success. (A) Comparison of Δcon from six core probes with different G/C content. For all probe sets, G/C content has been calculated as a mean value for all simulated probe lengths. (B) Diagram showing the linear dependence of probe length and G/C content for optimum specific probes after simulation and HSE extraction. Data points are based on Table 2. Diamonds represent the length and G/C content of probes with the highest simulated Δcon (n = 48), crosses indicate length and G/C content of the probe with the best HSE success (n = 11, grey boxes in Table 2A).

Finally, Δcon is presented as a function of probe length in Figure 5A. Here, Δcon appears as a parabola similar curve in which the maximum Δcon shifts to longer or shorter probe lengths depending on the G/C content. The amplitude of the maximum or shape of the curve is different between all probe sets and does not show any dependence on G/C content. Because of the partial dependency of Δcon on G/C content, the best predicted probes from all 41 tested probe sets were compared after G/C content and probe length. Figure 5B shows that the probe length at which Δcon reached its maximum decrease with increasing G/C content. When we plotted G/C content and the probe length of the most specific probes evaluated in HSE experiments (Figure 5B, crosses), meaning they were independent of the simulation, we obtained a similar linear dependency. Using linear regression (formula in Figure 5B), we could also predict optimal probe length by G/C content, which matched simulated and experimentally evaluated optimal probe length within only two nucleotides (data not shown).

## Discussion

Allele-specific extension was carried out using the protocol for HSE and tested for the separation of male DNA mixtures. Allele-specific probes were designed to detect Y chromosome SNPs that are proximal to forensically relevant STR markers. DNA mixtures were prepared by mixing two male samples that differ in selected SNP and STR markers. Probe designs had a base pair match on the 3′ end of the probe, allowing for the specific extension of the target allele by polymerase, which incorporates biotinylated nucleotides so that the intended Y chromosome haplotype can be separated from the mixture. We demonstrated the deep impact of optimal probe design and probe length on specific primer extension reactions.

Our results clearly demonstrate that length differences of only few base pairs can determine HSE efficiency. The probe we use as an example, P224 FC, demonstrated 63% separation efficiency when the HSE probe sequence was 23 nucleotides long, but when four nucleotides were removed from the 5′ end of the probe, 98% separation efficiency was observed. The dramatically improved separation of the two male samples with the 19-nucleotide HSE probe was unexpected, as shorter probes generally display lower sequence specificity for their targets and higher dissociation rates. This same effect was observed with a number of probes (Table 2). Though the 3′ termini were consistent in all evaluated probe sets, the improved separation with altered probe length was not likely caused by increased mismatch recognition by the polymerase. Because probe-associated factors, such as G/C content, melting temperature, and Gibb’s free energy (ΔG), were not determining factors that could be correlated to HSE efficiency, probe concentration between match and mismatch hybridization (Δcon_M-MM_) was compared. Comparing the simulated Δcon_M-MM_ values for each probe set with HSE separation success, we observed a strong correlation between maximum Δcon_M-MM_ and HSE efficiency. In more than 80% of the comprehensively tested probe sets, the length of the best HSE probes matched the best predicted probe length with no or only one base difference. These data show that Δcon between match and mismatch represents a potential prediction for probe specificity and indicates that a specific elongation of the 3′ end not only depends on the mismatch discrimination of the polymerase, but also on the ratio of available targets for match and mismatch. The relevance of the difference in concentration between the matched and mismatched probes in the HSE experiments is independent of target concentration, as no amplification of the target occurs in these reactions, and the probe concentrations are in the order of 10^9^ more concentrated than the intended genomic DNA targets. The data suggest that these concentration differences between probes and targets promote a highly competitive environment between match and mismatch probes in which small changes between probe concentrations apparently affect polymerase activity, as Δcon_M-MM_ is the only parameter demonstrated to predict HSE efficiency.

The curve progression of Δcon and HSE success did not always completely match. For example, for probe set P224FC, separation by HSE can still occur with very small Δcon values, downstream of the maximum Δcon, and the HSE curve of P30FA seems to be shifted to a probe shortened by one nucleotide. These minor changes could be caused by additional factors, such as short distance from the marker, favorable mismatch for polymerase discrimination, or a more specific sequence, even for shorter probe lengths.

Strong disagreement between the correlation of maximum Δcon and HSE success was observed mainly with P38. The simulated maximum Δcon was predicted to be much smaller than the length obtained for most specific probes after HSE experiments. A possible cause of this observation may be the high observed hit frequencies after the alignment of P38 in the 30 kb distance, which in turn might be caused by its high G/C contents. Guanosines are promiscuous and probes with low complexity that contain runs of three or more guanosines in a row tend to mishybridize [20]. Furthermore, the increased non-specific binding of P38 could cause rapid loss of the probe pool and influence final match and mismatch hybridization, which cannot be considered in the simulation. The simulation of P38 probe hybridization with lower starting concentration showed a switch of maximum Δcon to longer probe length and, therefore, could explain the observed results. Importantly, Δcon only provides information about the proportion of match and mismatch, which strongly influences polymerase discrimination in HSE but overlaps with polymerase specificity. Therefore, we conclude that the unexpected separation observed in HSE for probe rs13304202FA results from favorable mismatch recognition by the polymerase.

However, in this study we showed that the ability of the polymerase to discriminate between matched and mismatched probes was highest in 88% of the cases when the ratio of match to mismatch targets was at a maximum. Our data clearly prove that probes for specific extension reactions work only within a narrow and variable range of probe length. On one hand, the minimum probe length seems to be determined by several factors: a) decreasing probe length provokes less binding stability and reduced concentration in probe-target duplexes, b) with shorter probe length, the probe becomes more sequence non-specific, and c) Δcon between match and mismatch becomes insignificant.

It has to be considered that HSE includes the formation of huge homo- and hetero-duplexes on the genomic level. Therefore, hybridization temperature and time were set to 58°C and 20 minutes to allow rejoining of the complement strands, but this can also limit the available annealing temperature range for probe-specific binding. Furthermore, in HSE reactions, the entire human genome serves as a template for probe binding. Although a complete match of an average HSE probe (length 15–18 nt) is expected to occur only once in the entire human genome, the real number of matches can be increased dramatically through duplications, transpositions, and conserved sequence motifs in the genome. Therefore, binding sites for partially annealed probes occur in the genome for an unmanageable number, and due to the low annealing temperature of 58°C the concentration of non-specific binding strongly increases, whereas specific binding decreases with shorter probe length. On the other hand, the maximum probe length only seems to depend on the concentration difference between match and mismatch duplexes. When probe length is longer, mismatch hybridization occurs too often which could increase the failure of the mismatch discrimination by the polymerase.

In addition to the correlation of maximum Δcon and HSE success, we observed that the simulated Δcon partly depends on the G/C content of the probe and that increasing G/C content causes a shift of Δcon to shorter probe length. The same dependency of G/C content and probe length was observed for the best specific probes tested in HSE and confirms the simulated data for best probe length after match and mismatch hybridization with *Visual OMP*. Therefore our new prediction model provides an efficient, cost-effective method, which allows to design a better qualified pool of test-probes with less experimental optimization work.

## Supporting Information

File S1 **Methods.**
(DOC)

File S2 **Figure.**
(DOC)

File S3 **Datasets.**
(PDF)

File S4 **Figure.**
(DOC)
