# Supplementary material for: New Prediction Model for Probe Specificity in an Allele-Specific Extension Reaction for Haplotype-Specific Extraction (HSE) of Y Chromosome Mixtures
Source: PLoS One. 2012 Sep 25;7(9):e45955. doi: 10.1371/journal.pone.0045955 (PMC3457965; doi:10.1371/journal.pone.0045955)
Supplement: File S1 — Methods. (DOC) [file pone.0045955.s001.doc]

**Supplement 1**

**A Primer for Pyrosequencing**

**B Primer for Sanger sequencing**

**C Analysis of selected SNPs for probe design**

For both assays (Pyro- or Sanger sequencing) the primer design was based on the Basic Local Alignment Search Tool (Blast) of the amplified SNP region to avoid amplification of duplicated or homologous regions. OligoAnalyzer1.1.2 (http://oligo-analyzer.software.informer.com) and Oligo Explorer1.2 (www.genelink.com) were used to support the general primer design. Assay Design Software 1.0 (Biotage, Uppsala, Sweden) was applied especially for pyrosequencing primer design (Table 1).

All DNA amplification reactions used 1× PCR buffer; the 10× buffer contained 15 mM MgCl_2_, (Applied Biosystems, New Jersey, USA), 0.25 mM dNTPs (Roche, Mannheim, Germany), 0.5 µM of each primer (TIP MOLBIOL Berlin,), 1U AmpliTaq Gold ® Polymerase (Applied Biosystems, New Jersey, USA), and approximately 20 ng of genomic DNA. Prior to pyrosequencing, PCR amplifications were carried out in a 25 µl reaction with an additional 0.5 mM MgCl_2_ (Inviteck, Berlin, Germany) and one primer labeled with biotin (Table 1A). Prior to Sanger sequencing, PCR amplifications were carried out in a 10 µl reaction with unlabeled primers (Table 1B). Thermocycling parameters used for PCR prior to pyrosequencing were: 95°C for 10 min, followed by 8 cycles of: 95°C for 15 s, 60°C for 15 s, and 72°C for 30 s; then, 40 cycles of: 95°C for 15 s, 58°C for 15 s, and 72°C for 30 s; and finally, 70°C for 5 min. In the case of rs33963329, we used the following thermocycling parameters: 95°C for 10 min, followed by 40 cycles of: 95°C for 15 s, 54°C for 15 s, 72°C for 30 s; and finally, 70°C for 5 min. For Sanger sequencing, thermocycling included three steps; first, 95°C for 10 min, and 5 cycles of: 96°C for 20 s, 65°C for 45 s, and 72°C for 3 min; then, 20 cycles of: 96°C for 20 s, 60°C for 50 s, and 72°C for 3 min); and finally, 9 cycles of: 96°C for 20 s, 55°C for 1 min, and 72°C for 3 min. Prior to Sanger sequencing, the unused dNTPs were removed with ExoSAP-IT® for PCR product clean-up (USB, Cleveland, Ohio, USA). Therefore, 3 µl of ExoSAP-IT were added to PCR products and incubated for 30 min at 37°C. Reactions were stopped with an incubation for 15 min at 80°C.

Pyrosequencing has been carried out as described in (12)*.* The sequencing reaction was performed with the BigDye®Terminator (BDT) v1.1 Cycle Sequencing Kit (Applied Biosystems, Foster City, USA). Briefly, Ready Reaction Mix was diluted four times with BigDye sequencing buffer. The reaction was carried out in a 10 µl volume with 1 µl of BDT Ready Reaction Premix, 1.5 µl BDT BigDye sequencing buffer, 3 µl of the ExoSAP-IT treated PCR product, and 4.5 µl of water. The manufacturer’s parameters for double-stranded DNA were used for thermocycling. The sequencing product was purified by ethanol precipitation. Briefly, 2 µl of sodium acetate and 25 µl of absolute ethanol were added to the PCR product and centrifuged for 30 min at 2000 × g. Ethanol was removed by turning the tubes on filter paper and briefly centrifuging at 100 × g for 10 sec. Precipitated DNA was washed with 50 µl of 70% ethanol for 5 min at 2000 × g and, again, ethanol was removed (100 × g 10 sec). Samples were dried at room temperature for 15 min and resuspended in 15 µl of Hi-Di^TM^Formamide. Sequencing analysis was conducted according to the manufacturer’s settings on the ABI Prism®3130-Avant Genetic Analyzer in 36 cm capillaries filled with the denaturing polymer, POP-4 (Applied Biosystems, Foster City, USA). Data were collected with ABI Prism Data Collection Software 3.1. and processed with ABI Prism®Sequencing Analysis Software 5.2.
