# Supplementary material for: New Prediction Model for Probe Specificity in an Allele-Specific Extension Reaction for Haplotype-Specific Extraction (HSE) of Y Chromosome Mixtures
Source: PLoS One. 2012 Sep 25;7(9):e45955. doi: 10.1371/journal.pone.0045955 (PMC3457965; doi:10.1371/journal.pone.0045955)
Supplement: File S2 — Figure. (DOC) [file pone.0045955.s002.doc]

**Supplement 2.**


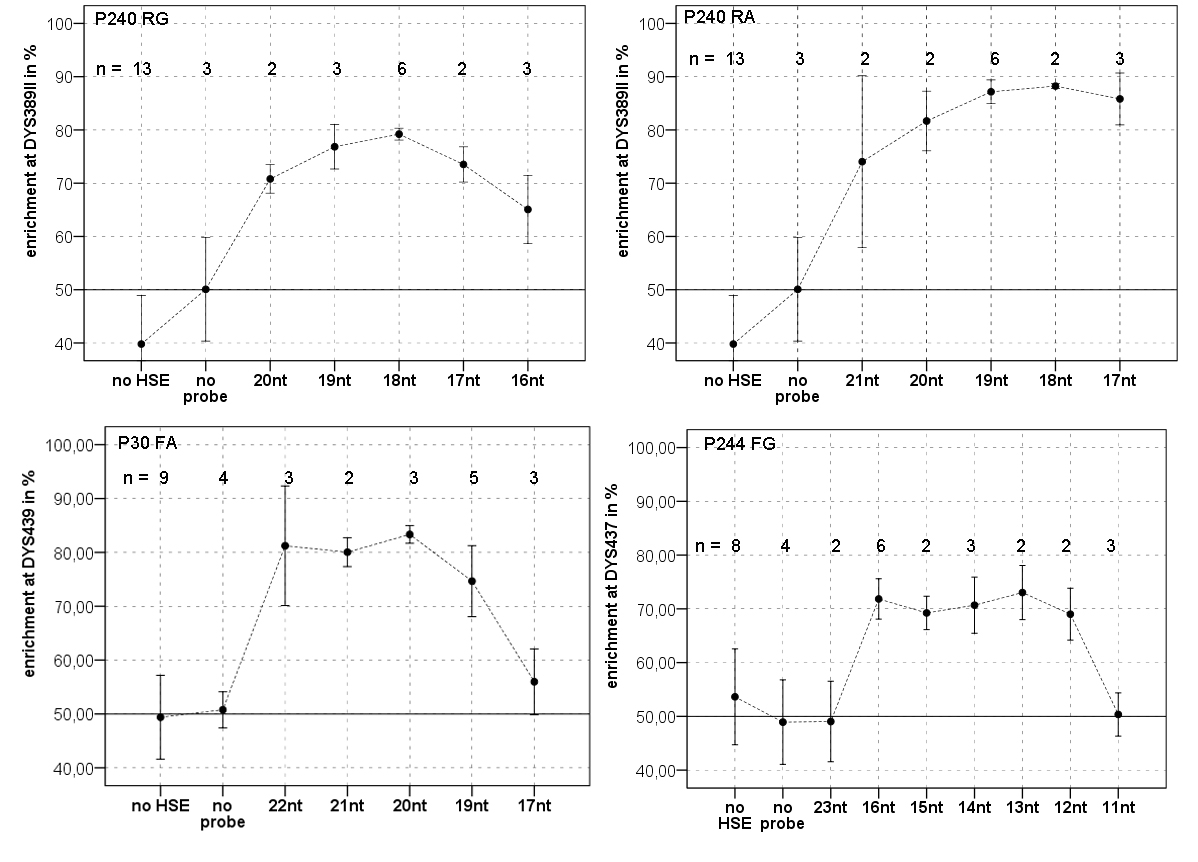
Separation by HSE with different probes. Enrichment of one contributor from a male DNA mixture is shown in dependence of probe length for 4 different probe sets P240RG/RA, P30FA and P244FG. Bars show standard deviation of mean enrichment, (no HSE) analysis of the male DNA mixture without separation by HSE, (no probe) separation by HSE of a male DNA mixture without probe, (n) number of extractions.
