# Supplementary material for: New Prediction Model for Probe Specificity in an Allele-Specific Extension Reaction for Haplotype-Specific Extraction (HSE) of Y Chromosome Mixtures
Source: PLoS One. 2012 Sep 25;7(9):e45955. doi: 10.1371/journal.pone.0045955 (PMC3457965; doi:10.1371/journal.pone.0045955)
Supplement: File S3 — Datasets. (PDF) [file pone.0045955.s003.pdf]

probe	l	sequence	GC%	Tm NN	ΔG_M	ΔG_{MM}	$\Delta\Delta G_{M\text{-}MM}$	$con_M \cdot 10^{-17}M$	$con_{MM} \cdot 10^{-17}M$	$\Delta con_{M\text{-}MM} \cdot 10^{-17}M$	HSE result in %	SD	n
P30 FG - DYS 439	**24**	GACAGGTGATAGATAAGTTGATCG	41	63	-11,11	-9,96	-1,15	297	285	**12**	**-**		
	**23**	ACAGGTGATAGATAAGTTGATCG	39	63	-10,74	-9,59	-1,15	295	274	**21**	-		
	**22**	CAGGTGATAGATAAGTTGATCG	40	61	-9,51	-8,36	-1,15	271	187	**85**	**60**		1
	**21**	AGGTGATAGATAAGTTGATCG	38	60	-9,05	-7,90	-1,15	247	135	**112**	**76**	1	2
	**20**	GGTGATAGATAAGTTGATCG	40	58	-8,16	-7,01	-1,15	165	52	**112**	**70**		1
	**19**	GTGATAGATAAGTTGATCG	36	55	-6,90	-5,70	-1,20	43	8	**34**	**75**	8	4
	**18**	TGATAGATAAGTTGATCG	33	53	-5,91	-4,76	-1,15	11	2	**9**	-		
	**17**	GATAGATAAGTTGATCG	35	52	-5,60	-4,41	-1,19	7	1	**6**	**63**	19	2
	**16**	ATAGATAAGTTGATCG	31	50	-4,92	-3,87	-1,05	3	1	**2**	-		
	**15**	TAGATAAGTTGATCG	33	47	-4,23	-3,18	-1,05	1	0	**1**	-		
	**14**	AGATAAGTTGATCG	35	49	-4,83	-3,78	-1,05	2	0	**2**	**-**		
P30 FG-1 - DYS 439	**25**	GACAGGTGATAGATAAGTTGATCGA	40	64	-11,77	-9,96	-1,81	299	285	**14**			
	**24**	ACAGGTGATAGATAAGTTGATCGA	38	64	-11,4	-9,59	-1,81	298	274	**24**			
	**23**	CAGGTGATAGATAAGTTGATCGA	39	62	-10,17	-8,36	-1,81	289	187	**102**	-		
	**22**	AGGTGATAGATAAGTTGATCGA	36	61	-9,71	-7,90	-1,81	278	135	**143**	-		
	**21**	GGTGATAGATAAGTTGATCGA	38	60	-8,81	-7,01	-1,80	230	52	**177**	**82**	3	4
	**20**	GTGATAGATAAGTTGATCGA	35	57	-7,51	-5,70	-1,81	93	8	**85**	-		
	**19**	TGATAGATAAGTTGATCGA	32	55	-6,57	-4,76	-1,81	29	2	**27**	-		
	**18**	GATAGATAAGTTGATCGA	33	54	-6,22	-4,41	-1,81	18	1	**17**	-		
	**17**	ATAGATAAGTTGATCGA	30	52	-5,57	-3,87	-1,70	7	1	**6**	-		
	**16**	TAGATAAGTTGATCGA	31	49	-4,79	-3,18	-1,61	2	0	**2**	-		
	**15**	AGATAAGTTGATCGA	33	51	-5,38	-3,78	-1,60	5	0	**5**	**-**		
P30 FA - DYS 439	**40**	TAATAGATAGATGATAGACAGGTGATAGATAAGTTGATCA	30	67	-16,14	-15,74	-0,40	300	300	**0**	**-**		
	**35**	GATAGATGATAGACAGGTGATAGATAAGTTGATCA	34	67	-15,18	-14,78	-0,40	300	300	**0**	-		
	**30**	ATGATAGACAGGTGATAGATAAGTTGATCA	33	66	-13,22	-12,82	-0,40	300	300	**0**	-		
	**28**	GATAGACAGGTGATAGATAAGTTGATCA	36	64	-12,1	-11,7	-0,40	299	299	**1**	-		
	**26**	TAGACAGGTGATAGATAAGTTGATCA	35	63	-10,57	-10,17	-0,40	294	289	**5**	-		
	**25**	AGACAGGTGATAGATAAGTTGATCA	36	64	-11,16	-10,76	-0,40	297	295	**2**	-		
	**24**	GACAGGTGATAGATAAGTTGATCA	38	62	-10,23	-9,83	-0,40	290	282	**8**	-		
	**23**	ACAGGTGATAGATAAGTTGATCA	35	62	-9,86	-9,46	-0,40	282	269	**13**	-		
	**22**	CAGGTGATAGATAAGTTGATCA	36	59	-8,63	-8,23	-0,40	214	172	**41**	**76**	5	3
	**21**	AGGTGATAGATAAGTTGATCA	33	58	-8,17	-7,77	-0,40	166	120	**45**	**80**	3	2
	**20**	GGTGATAGATAAGTTGATCA	35	56	-7,28	-6,88	-0,40	73	44	**28**	**83**	2	3
	**19**	GTGATAGATAAGTTGATCA	32	53	-5,97	-5,57	-0,40	13	7	**6**	**75**	6	5
	**18**	TGATAGATAAGTTGATCA	28	51	-5,03	-4,63	-0,40	3	2	**1**	-		
	**17**	GATAGATAAGTTGATCA	29	50	-4,68	-4,28	-0,40	2	1	**1**	**56**	6	3
	**16**	ATAGATAAGTTGATCA	25	47	-4,04	-3,74	-0,30	1	0	**0**	-		
	**15**	TAGATAAGTTGATCA	27	44	-3,35	-3,05	-0,30	0	0	**0**	-		
	**14**	AGATAAGTTGATCA	29	46	-3,95	-3,65	-0,30	1	0	**0**	-		
	**13**	GATAAGTTGATCA	31	43	-3,11	-2,82	-0,29	0	0	**0**	-		
	**12**	ATAAGTTGATCA	25	38	-2,84	-2,55	-0,29	0	0	**0**	-		
	**11**	TAAGTTGATCA	27	33	-2,16	-1,86	-0,30	0	0	**0**	-		
	**10**	AAGTTGATCA	30	36	-2,76	-2,46	-0,30	0	0	**0**	-		
	**8**	GTTGATCA	38	27	-1,66	-1,36	-0,30	0	0	**0**	-		
	**6**	TGATCA	33	7	-0,58	-0,28	-0,30	0	0	**0**	**-**		

P30 FA-1 - DYS 439	25	GACAGGTGATAGATAAGTTGATCAA	36	63	-10,84	-9,83	-1,01	296	282	14	-		
	24	ACAGGTGATAGATAAGTTGATCAA	33	63	-10,47	-9,46	-1,01	293	269	24	-		
	23	CAGGTGATAGATAAGTTGATCAA	35	60	-9,24	-8,23	-1,01	259	172	86	-		
	22	AGGTGATAGATAAGTTGATCAA	32	60	-8,78	-7,77	-1,01	227	120	107	95	2	2
	21	GGTGATAGATAAGTTGATCAA	33	58	-7,89	-6,88	-1,01	134	44	89	84	5	3
	20	GTGATAGATAAGTTGATCAA	30	55	-6,58	-5,57	-1,01	30	7	23	-		
	19	TGATAGATAAGTTGATCAA	26	52	-5,64	-4,63	-1,01	8	2	6	-		
	18	GATAGATAAGTTGATCAA	28	52	-5,29	-4,28	-1,01	5	1	4	-		
	17	ATAGATAAGTTGATCAA	24	49	-4,65	-3,74	-0,91	2	0	1	-		
	16	TAGATAAGTTGATCAA	25	46	-3,86	-3,05	-0,81	1	0	0	-		
P30 RC - DYS 439	26	TTCTATCCATCTATCATCTATTTATC	27	57	-7,43	-6,94	-0,49	86	48	38	-		
	25	TCTATCCATCTATCATCTATTTATC	28	57	-7,55	-7,06	-0,49	97	56	42	86	0	2
	24	CTATCCATCTATCATCTATTTATC	29	57	-7,63	-7,14	-0,49	106	61	44	92	9	2
	23	TATCCATCTATCATCTATTTATC	26	54	-6,02	-5,53	-0,49	13	7	7	-		
	22	ATCCATCTATCATCTATTTATC	27	55	-6,62	-6,13	-0,49	31	16	16	51	1	2
	21	TCCATCTATCATCTATTTATC	28	53	-5,47	-4,98	-0,49	6	3	3	-		
	20	CCATCTATCATCTATTTATC	30	54	-6,04	-5,55	-0,49	14	7	7	-		
	19	CATCTATCATCTATTTATC	26	49	-4,14	-3,66	-0,48	1	0	0	46	8	2
	18	ATCTATCATCTATTTATC	22	47	-3,56	-3,07	-0,49	0	0	0			

P30 RC-1 - DYS 439	28	ATTCTATCCATCTATCATCTATTTATCG	29	91	-9,7	-7,52	-2,18	278	94	184			
	27	TTCTATCCATCTATCATCTATTTATCG	30	60	-9,12	-6,94	-2,18	252	48	204	-		
	26	TCTATCCATCTATCATCTATTTATCG	31	60	-9,24	-7,06	-2,18	259	56	203	70	2	3
	25	CTATCCATCTATCATCTATTTATCG	32	60	-9,32	-7,14	-2,18	263	61	201	-		
	24	TATCCATCTATCATCTATTTATCG	30	57	-7,71	-5,53	-2,18	114	7	107	-		
	23	ATCCATCTATCATCTATTTATCG	30	59	-8,31	-6,13	-2,18	181	16	165	73	4	3
	22	TCCATCTATCATCTATTTATCG	32	56	-7,16	-4,98	-2,18	63	3	60	-		
	21	CCATCTATCATCTATTTATCG	33	57	-7,73	-5,55	-2,18	116	7	109	-		
	20	CATCTATCATCTATTTATCG	30	53	-5,83	-3,66	-2,17	10	0	10	-		
	19	ATCTATCATCTATTTATCG	26	52	-5,25	-3,07	-2,18	4	0	4	-		

probe	l	sequence	GC %	Tm NN	ΔG_M	ΔG_{MM}	$\Delta\Delta G_{M-MM}$	con_M* 10^{-17}M	con_{MM}* 10^{-17}M	Δcon_{MM}* 10^{-17}M	HSE result in %	SD	n
P38 FA - DYS 437	**40**	AGCAGTCTGCCCTGTGGGGGCTGGGAGGGTGGCTCCCGCA	73	90	-37,29	-36,69	-0,60	300	300	**0**	**-**		
	**35**	TCTGCCCTGTGGGGGCTGGGAGGGTGGCTCCCGCA	74	89	-32,36	-31,76	-0,60	300	300	**0**	-		
	**30**	CCTGTGGGGGCTGGGAGGGTGGCTCCCGCA	77	86	-27,58	-26,98	-0,60	300	300	**0**	-		
	**28**	TGTGGGGGCTGGGAGGGTGGCTCCCGCA	75	84	-25,17	-24,57	-0,60	300	300	**0**	-		
	**26**	TGGGGGCTGGGAGGGTGGCTCCCGCA	77	85	-23,85	-23,25	-0,60	300	300	**0**	-		
	**25**	GGGGGCTGGGAGGGTGGCTCCCGCA	80	84	-23,50	-22,89	-0,61	300	300	**0**	-		
	**24**	GGGGCTGGGAGGGTGGCTCCCGCA	79	83	-21,92	-21,32	-0,60	300	300	**0**	-		
	**23**	GGGCTGGGAGGGTGGCTCCCGCA	78	81	-20,66	-20,06	-0,60	300	300	**0**	-		
	**22**	GGCTGGGAGGGTGGCTCCCGCA	77	80	-19,40	-18,80	-0,60	300	300	**0**	-		
	**21**	GCTGGGAGGGTGGCTCCCGCA	76	78	-18,14	-17,54	-0,60	300	300	**0**	**89**		1
	**20**	CTGGGAGGGTGGCTCCCGCA	75	77	-16,66	-16,06	-0,60	300	300	**0**	**87**		1
	**19**	TGGGAGGGTGGCTCCCGCA	74	74	-15,49	-14,88	-0,61	300	300	**0**	**85**	6	2
	**18**	GGGAGGGTGGCTCCCGCA	78	75	-15,42	-14,82	-0,60	300	300	**0**	-		
	**17**	GGAGGGTGGCTCCCGCA	76	72	-13,84	-13,24	-0,60	300	300	**0**	-		
	**16**	GAGGGTGGCTCCCGCA	75	70	-12,58	-12,08	-0,50	300	299	**0**	**67**		1
	**15**	AGGGTGGCTCCCGCA	73	71	-12,36	-11,86	-0,50	300	299	**0**	**68**	6	3
	**14**	GGGTGGCTCCCGCA	79	68	-11,49	-10,99	-0,50	298	297	**2**	-		
	**13**	GGTGGCTCCCGCA	77	65	-10,29	-9,79	-0,50	291	281	**10**	**54**		1
	**12**	GTGGCTCCCGCA	75	62	-9,13	-8,63	-0,50	252	214	**39**	**50**		1
	**11**	TGGCTCCCGCA	73	59	-8,29	-7,79	-0,50	179	123	**56**	**46**		1
	**10**	GGCTCCCGCA	80	58	-8,04	-7,54	-0,50	151	96	**55**	**50**	5	2
	**8**	CTCCCGCA	75	30	-5,18	-4,68	-0,50	4	2	**2**	-		
	**6**	CCCGCA	83	37	-4,65	-4,15	-0,50	2	1	**1**	**-**		
P38 FA-1 - DYS 437	**20**	TGGGAGGGTGGCTCCCGCAT	70	75	-15,93	-14,88	-1,05	300	300	**0**	**-**		
	**19**	GGGAGGGTGGCTCCCGCAT	73	76	-15,86	-14,82	-1,04	300	300	**0**	**86**		1
	**18**	GGAGGGTGGCTCCCGCAT	72	73	-14,28	-13,24	-1,04	300	300	**0**	-		
	**17**	GAGGGTGGCTCCCGCAT	71	71	-13,02	-12,08	-0,94	300	299	**0**	-		
	**16**	AGGGTGGCTCCCGCAT	69	71	-12,70	-11,86	-0,84	300	299	**1**	**79**		1
	**15**	GGGTGGCTCCCGCAT	73	69	-11,83	-10,99	-0,84	299	297	**2**	-		
	**14**	GGTGGCTCCCGCAT	71	66	-10,63	-9,79	-0,84	294	281	**14**	-		
	**13**	GTGGCTCCCGCAT	68	63	-9,47	-8,63	-0,84	270	214	**56**	-		
	**12**	TGGCTCCCGCAT	67	60	-8,63	-7,79	-0,84	214	123	**91**	**50**		1
	**11**	GGCTCCCGCAT	73	59	-8,38	-7,54	-0,84	189	96	**92**	**-**		
P38 FC - DYS 437	**20**	CTGGGAGGGTGGCTCCCGCC	80	78	-17,14	-15,90	-1,24	300	300	**0**	**60**		1
	**19**	TGGGAGGGTGGCTCCCGCC	78	75	-15,97	-14,73	-1,24	300	300	**0**	**60**		1
	**18**	GGGAGGGTGGCTCCCGCC	83	76	-15,90	-14,66	-1,24	300	300	**0**	**70**	9	4
	**17**	GGAGGGTGGCTCCCGCC	82	73	-14,32	-13,08	-1,24	300	300	**0**	-		
	**16**	GAGGGTGGCTCCCGCC	81	71	-13,07	-11,93	-1,14	300	299	**1**	-		
	**15**	AGGGTGGCTCCCGCC	80	72	-12,90	-11,71	-1,19	300	299	**1**	**72**	3	5
	**14**	GGGTGGCTCCCGCC	85	69	-11,98	-10,84	-1,14	299	296	**3**	-		
	**13**	GGTGGCTCCCGCC	85	67	-10,77	-9,63	-1,14	295	276	**20**	**60**		1
	**12**	GTGGCTCCCGCC	83	63	-9,61	-8,47	-1,14	275	198	**77**	**59**		1
	**11**	TGGCTCCCGCC	81	57	-7,79	-7,64	-0,15	226	107	**120**	-		
	**10**	GGCTCCCGCC	90	56	-7,54	-7,39	-0,15	203	82	**121**	**50**		1
	**9**	GCTCCCGCC	89	48	-7,05	-5,91	-1,14	55	11	**44**	-		

	**8**	CTCCCGCC	88	46	-5,66	-4,52	-1,14	8	1	**7**	**-**		

P38 FC-1 - DYS 437	**19**	GGGAGGGTGGCTCCCGCCT	79	78	-16,70	-14,66	-2,04	300	300	**0**	**-**		
	**18**	GGAGGGTGGCTCCCGCCT	78	75	-15,12	-13,08	-2,04	300	300	**0**	**49**	4	3
	**17**	GAGGGTGGCTCCCGCCT	77	73	-13,86	-11,93	-1,93	300	299	**1**	-		
	**16**	AGGGTGGCTCCCGCCT	75	74	-13,54	-11,71	-1,83	300	299	**1**	-		
	**15**	GGGTGGCTCCCGCCT	80	71	-12,67	-10,84	-1,83	300	296	**4**	-		
	**14**	GGTGGCTCCCGCCT	89	68	-11,46	-9,63	-1,83	298	276	**23**	-		
	**13**	GTGGCTCCCGCCT	77	65	-10,31	-8,47	-1,84	291	198	**93**	-		
	**12**	TGGCTCCCGCCT	75	63	-9,47	-7,64	-1,83	270	107	**163**	**39**		1
	**11**	GGCTCCCGCCT	81	62	-9,22	-7,39	-1,83	258	82	**176**	**-**		

probe	l	sequence	GC %	Tm NN	ΔG_M	ΔG_{MM}	$\Delta\Delta G_{M-MM}$	con_M* 10^{-17}M	con_{MM}* 10^{-17}M	Δcon_{M-MM}* 10^{-17}M	HSE result in %	SD	n
	**40**	TACTCTCAGAAATAATTTCAGAAATGAGTGTGACATCTTC	33	70	-18,71	-17,98	-0,73	300	300	**0**	-		
	**39**	ACTCTCAGAAATAATTTCAGAAATGAGTGTGACATCTTC	33	70	-19,11	-18,38	-0,73	300	300	**0**	-		
	**35**	TCAGAAATAATTTCAGAAATGAGTGTGACATCTTC	31	67	-15,56	-14,83	-0,73	300	300	**0**	-		
	**34**	CAGAAATAATTTCAGAAATGAGTGTGACATCTTC	32	68	-16,00	-15,27	-0,73	300	300	**0**	-		
	**33**	AGAAATAATTTCAGAAATGAGTGTGACATCTTC	30	67	-14,78	-14,05	-0,73	300	300	**0**	-		
	**32**	GAAATAATTTCAGAAATGAGTGTGACATCTTC	31	66	-13,88	-13,15	-0,73	300	300	**0**	-		
	**31**	AAATAATTTCAGAAATGAGTGTGACATCTTC	29	66	-13,51	-12,78	-0,73	300	300	**0**	-		
	**30**	AATAATTTCAGAAATGAGTGTGACATCTTC	30	66	-13,15	-12,42	-0,73	300	300	**0**	-		
	**29**	ATAATTTCAGAAATGAGTGTGACATCTTC	31	65	-12,78	-12,05	-0,73	300	299	**0**	-		
	**28**	TAATTTCAGAAATGAGTGTGACATCTTC	32	64	-11,89	-11,16	-0,73	299	297	**2**	-		
	**27**	AATTTCAGAAATGAGTGTGACATCTTC	33	65	-12,49	-11,76	-0,73	300	299	**1**	-		
	**26**	ATTTCAGAAATGAGTGTGACATCTTC	35	65	-12,16	-11,43	-0,73	299	298	**1**	-		
	**25**	TTTCAGAAATGAGTGTGACATCTTC	36	63	-10,97	-10,24	-0,73	297	290	**7**	-		
	**24**	TTCAGAAATGAGTGTGACATCTTC	38	63	-11,09	-10,36	-0,73	297	292	**6**	-		
P224FC - DYS390	**23**	TCAGAAATGAGTGTGACATCTTC	39	63	-10,73	-9,99	-0,74	295	285	**10**	**68**	11	4
	**22**	CAGAAATGAGTGTGACATCTTC	41	63	-10,80	-10,07	-0,73	0	0	**0**	-		
	**21**	AGAAATGAGTGTGACATCTT**C**	38	61	-9,58	-8,85	-0,73	274	233	**41**	**85**	4	3
	**20**	GAAATGAGTGTGACATCTT**C**	40	59	-8,69	-7,95	-0,74	219	141	**79**	**91**	5	6
	**19**	AAATGAGTGTGACATCTT**C**	37	59	-8,32	-7,58	-0,74	182	100	**82**	**89**	5	10
	**18**	AATGAGTGTGACATCTT**C**	39	58	-7,95	-7,22	-0,73	141	68	**73**	**91**	7	7
	**17**	ATGAGTGTGACATCTT**C**	41	57	-7,58	-6,85	-0,73	100	43	**58**	**88**	9	8
	**16**	TGAGTGTGACATCTT**C**	44	54	-6,40	-5,77	-0,63	23	9	**14**	**91**	7	7
	**15**	GAGTGTGACATCTT**C**	47	54	-6,56	-5,93	-0,63	29	12	**17**	**92**	4	6
	**14**	AGTGTGACATCTT**C**	43	52	-6,02	-5,39	-0,63	13	5	**8**	**86**	6	6
	**13**	GTGTGACATCTT**C**	46	49	-5,15	-4,52	-0,63	4	1	**2**	**84**	6	5
	**12**	TGTGACATCTT**C**	42	45	-4,26	-3,63	-0,63	1	0	**1**	**70**	5	3
	**11**	GTGACATCTT**C**	45	44	-4,01	-3,38	-0,63	1	0	**0**	**79**	5	3
	**10**	TGACATCTT**C**	40	36	-2,85	-2,22	-0,63	0	0	**0**	**57**	6	3
	**9**	GACATCTT**C**	44	35	-2,60	-1,97	-0,63	0	0	**0**	**54**	7	3
	**8**	ACATCTTC	38	26	-2,06	-1,43	-0,63	0	0	**0**	-		
	**6**	CATCTTC	43	21	-0,93	-0,32	-0,61	0	0	**0**	**-**		
	**25**	TTTCAGAAATGAGTGTGACATCTT**T**	32	63	-10,92	-10,22	-0,70	296	290	**7**	**-**		
	**24**	TTCAGAAATGAGTGTGACATCTT**T**	33	63	-11,05	-10,34	-0,71	297	291	**6**	-		
	**23**	TCAGAAATGAGTGTGACATCTT**T**	35	63	-10,68	-9,98	-0,70	295	285	**10**	**60**	9	5
P224FT - DYS390	**22**	CAGAAATGAGTGTGACATCTT**T**	36	63	-10,76	-10,05	-0,71	295	287	**9**	-		
	**21**	AGAAATGAGTGTGACATCTT**T**	33	61	-9,53	-8,83	-0,70	272	231	**41**	**75**	5	3
	**20**	GAAATGAGTGTGACATCTT**T**	35	59	-8,64	-7,94	-0,70	215	139	**75**	**84**	6	5
	**19**	AAATGAGTGTGACATCTT**T**	31	59	-8,27	-7,57	-0,70	177	99	**77**	**86**	5	3
	**18**	AATGAGTGTGACATCTT**T**	33	58	-7,91	-7,20	-0,71	136	66	**70**	**78**	6	3
	**17**	ATGAGTGTGACATCTT**T**	35	57	-7,54	-6,84	-0,70	96	42	**54**	-		
	**16**	TGAGTGTGACATCTT**T**	38	54	-6,35	-5,75	-0,60	22	9	**13**	**-**		
P224RG - DYS390	**20**	GTGGTTTCAGTCAGCAGGG**G**	60	68	-13,00	-11,71	-1,29	300	299	**1**	**78**		1
	**19**	TGGTTTCAGTCAGCAGGG**G**	58	67	-12,03	-10,72	-1,31	299	295	**4**	-		
	**18**	GGTTTCAGTCAGCAGGG**G**	61	66	-11,68	-10,37	-1,31	299	292	**7**	-		
	**17**	GTTTCAGTCAGCAGGG**G**	58	63	-10,10	-8,79	-1,31	288	228	**60**	**93**		1

	**16**	TTTCAGTCAGCAGGG**G**	56	61	-9,16	-7,95	-1,21	254	141	**114**	-	
	**15**	TTCAGTCAGCAGGG**G**	60	61	-8,97	-7,76	-1,21	242	119	**122**	-	
P224RG	**14**	TCAGTCAGCAGGG**G**	64	60	-8,70	-7,49	-1,21	220	91	**129**	**-**	
-	**13**	CAGTCAGCAGGG**G**	69	61	-8,88	-7,67	-1,23	235	110	**125**		
DYS390	**12**	AGTCAGCAGGG**G**	67	57	-7,76	-6,55	-1,25	119	28	**91**		

	**20**	GTGGTTTCAGTCAGCAGGG**A**	55	67	-12,20	-11,70	-0,50	299	299	**1**	**66**	1
	**19**	TGGTTTCAGTCAGCAGGG**A**	52	65	-11,19	-10,71	-0,48	298	295	**3**	-	
P224RA	**18**	GGTTTCAGTCAGCAGGG**A**	55	65	-10,84	-10,36	-0,48	296	292	**4**	-	
-	**17**	GTTTCAGTCAGCAGGG**A**	52	61	-9,26	-8,78	-0,48	260	227	**33**	**86**	1
DYS390	**16**	TTTCAGTCAGCAGGG**A**	50	59	-8,32	-7,94	-0,38	182	139	**43**	-	
	**15**	TTCAGTCAGCAGGG**A**	53	58	-8,13	-7,75	-0,38	161	118	**43**	-	
	**14**	TCAGTCAGCAGGG**A**	57	58	-7,86	-7,49	-0,37	130	91	**39**	**-**	

probe	l	sequence	GC%	Tm NN	ΔG_M	ΔG_{MM}	$\Delta\Delta G_{M-MM}$	$con_M * 10^{-17} M$	$con_{MM} * 10^{-17} M$	$\Delta con_{M-MM} * 10^{-17} M$	HSE result in %	SD	n
P240 FC - DYS 389II	23	ATCTTTCAGATCAATAACGTCTC	34	61	-9,72	-9,23	-0,49	279	258	20	-		
	22	TCTTTCAGATCAATAACGTCTC	36	59	-8,53	-8,05	-0,48	204	152	52	82		1
	21	CTTTCAGATCAATAACGTCTC	38	60	-9,10	-8,61	-0,49	251	212	39	-		
	20	TTTCAGATCAATAACGTCTC	35	57	-7,32	-6,83	-0,49	76	42	34	-		
	19	TTCAGATCAATAACGTCTC	36	56	-7,32	-6,83	-0,49	76	42	34	75	5	2
	18	TCAGATCAATAACGTCTC	38	56	-6,95	-6,46	-0,49	49	25	23	-		
	17	CAGATCAATAACGTCTC	41	56	-7,03	-6,54	-0,49	54	28	26	58		1
	16	AGATCAATAACGTCTC	37	52	-5,80	-5,41	-0,39	10	5	4	-		
P240 FC-1 - DYS 389II	24	ATCTTTCAGATCAATAACGTCTCG	37	65	-11,66	-9,23	-2,43	299	258	41	-		
	23	TCTTTCAGATCAATAACGTCTCG	39	62	-10,47	-8,05	-2,42	293	152	141	-		
	22	CTTTCAGATCAATAACGTCTCG	41	64	-11,04	-8,61	-2,43	297	212	85	-		
	21	TTTCAGATCAATAACGTCTCG	38	60	-9,26	-6,83	-2,43	260	42	218	82		1
	20	TTCAGATCAATAACGTCTCG	40	61	-9,26	-6,83	-2,43	260	42	218	81		1
	19	TCAGATCAATAACGTCTCG	42	60	-8,89	-6,46	-2,43	236	25	211	83	2	3
	18	CAGATCAATAACGTCTCG	44	60	-8,97	-6,54	-2,43	242	28	214	79		1
	17	AGATCAATAACGTCTCG	41	57	-7,74	-5,41	-2,33	117	5	112	-		
P240 FT - DYS 389II	23	ATCTTTCAGATCAATAACGTCTT	34	62	-9,82	-9,21	-0,61	281	257	24	-		
	22	TCTTTCAGATCAATAACGTCTT	31	59	-8,63	-8,03	-0,60	214	150	64	84	5	2
	21	CTTTCAGATCAATAACGTCTT	33	60	-9,20	-8,60	-0,60	257	211	46	-		
	20	TTTCAGATCAATAACGTCTT	30	57	-7,42	-6,82	-0,60	85	41	44	-		
	19	TTCAGATCAATAACGTCTT	32	57	-7,41	-6,81	-0,60	84	40	43	-		
	18	TCAGATCAATAACGTCTT	33	56	-7,04	-6,44	-0,60	54	25	30	-		
	17	CAGATCAATAACGTCTT	35	56	-7,12	-6,52	-0,60	60	27	33	-		
	16	AGATCAATAACGTCTT	31	52	-5,90	-5,40	-0,50	11	5	6	-		
P240 FT-1 - DYS 389II	24	ATCTTTCAGATCAATAACGTCTTG	33	63	-10,73	-9,21	-1,52	295	257	38	-		
	23	TCTTTCAGATCAATAACGTCTTG	35	61	-9,55	-8,03	-1,52	273	150	123	-		
	22	CTTTCAGATCAATAACGTCTTG	36	62	-10,12	-8,60	-1,52	288	211	77	-		
	21	TTTCAGATCAATAACGTCTTG	33	59	-8,34	-6,82	-1,52	184	41	143	86		1
	20	TTCAGATCAATAACGTCTTG	35	59	-8,33	-6,81	-1,52	183	40	143	94	5	3
	19	TCAGATCAATAACGTCTTG	37	58	-7,96	-6,44	-1,52	142	25	117	-		
	18	CAGATCAATAACGTCTTG	39	58	-8,04	-6,52	-1,52	151	27	123	-		
	17	AGATCAATAACGTCTTG	35	55	-6,82	-5,40	-1,42	41	5	36	-		
P240 RG - DYS 389II	21	GGTAGGCTCAGATAAAGAACG	47	64	-10,91	-9,77	-1,14	296	280	16	-		
	20	GTAGGCTCAGATAAAGAACG	45	61	-9,34	-8,19	-1,15	264	168	96	71	3	2
	19	TAGGCTCAGATAAAGAACG	42	59	-8,40	-7,25	-1,15	191	70	121	77	4	3
	18	AGGCTCAGATAAAGAACG	44	60	-8,87	-7,73	-1,14	234	116	118	79	1	6
	17	GGCTCAGATAAAGAACG	47	58	-7,94	-6,79	-1,15	139	39	100	74	4	2
	16	GCTCAGATAAAGAACG	43	55	-6,63	-5,59	-1,04	32	7	25	65	7	3
	15	CTCAGATAAAGAACG	40	50	-5,25	-4,20	-1,05	4	1	3	-		
	14	TCAGATAAAGAACG	35	47	-4,18	-3,13	-1,05	1	0	1	-		
	13	CAGATAAAGAACG	38	48	-4,72	-3,67	-1,05	2	0	2	-		
	22	GGTAGGCTCAGATAAAGAACGA	45	64	-10,98	-9,77	-1,21	297	280	17	-		

P240 RG-1 - DYS 389II	21	GTAGGCTCAGATAAAGAACGA	43	61	-9,40	-8,19	-1,21	267	168	**99**	-		
	20	TAGGCTCAGATAAAGAACGA	40	59	-8,46	-7,25	-1,21	197	70	**127**	**73**	1	2
	19	AGGCTCAGATAAAGAACGA	42	60	-8,94	-7,73	-1,21	240	116	**124**	**73**	3	2
	18	GGCTCAGATAAAGAACGA	44	58	-8,00	-6,79	-1,21	146	39	**107**	-		
	17	GCTCAGATAAAGAACGA	41	55	-6,70	-5,59	-1,11	35	7	**28**	-		
	16	CTCAGATAAAGAACGA	38	51	-5,21	-4,20	-1,01	4	1	**3**	-		
	15	TCAGATAAAGAACGA	33	48	-4,14	-3,13	-1,01	1	0	**1**	-		
	14	CAGATAAAGAACGA	36	48	-4,68	-3,67	-1,01	2	0	**1**	-		

P240 RA - DYS 389II	40	AGTGAAGGAGGGGCTGGCTGGTAGGCTCAGATAAAGAACA	53	80	-27,74	-27,34	-0,40	300	300	**0**	-		
	35	AGGAGGGGCTGGCTGGTAGGCTCAGATAAAGAACA	54	79	-24,46	-24,06	-0,40	300	300	**0**	-		
	30	GGGCTGGCTGGTAGGCTCAGATAAAGAACA	53	75	-19,57	-19,18	-0,39	300	300	**0**	-		
	28	GCTGGCTGGTAGGCTCAGATAAAGAACA	50	72	-17,06	-16,66	-0,40	300	300	**0**	-		
	24	GCTGGTAGGCTCAGATAAAGAACA	46	67	-12,76	-12,36	-0,40	300	300	**0**	-		
	23	CTGGTAGGCTCAGATAAAGAACA	43	64	-11,27	-10,87	-0,40	298	296	**2**	-		
	22	TGGTAGGCTCAGATAAAGAACA	41	62	-10,10	-9,70	-0,40	288	278	**10**	-		
	21	GGTAGGCTCAGATAAAGAACA	43	62	-10,03	-9,63	-0,40	286	276	**11**	**66**	6	2
	20	GTAGGCTCAGATAAAGAACA	40	59	-8,45	-8,06	-0,39	196	153	**43**	**82**	6	2
	19	TAGGCTCAGATAAAGAACA	37	57	-7,51	-7,12	-0,39	93	60	**33**	**87**	2	6
	18	AGGCTCAGATAAAGAACA	39	58	-7,99	-7,59	-0,40	145	101	**44**	**88**	1	2
	17	GGCTCAGATAAAGAACA	41	56	-7,06	-6,66	-0,40	56	33	**23**	**86**	5	3
	16	GCTCAGATAAAGAACA	38	52	-5,75	-5,45	-0,30	9	6	**3**	-		
	15	CTCAGATAAAGAACA	33	48	-4,37	-4,07	-0,30	1	1	**0**	-		
	14	TCAGATAAAGAACA	29	44	-3,30	-3,00	-0,30	0	0	**0**	-		
	13	CAGATAAAGAACA	31	44	-3,84	-3,54	-0,30	1	0	**0**	-		
	12	AGATAAAGAACA	25	39	-2,71	-2,42	-0,29	0	0	**0**	-		
	11	GATAAAGAACA	27	36	-1,92	-1,62	-0,30	0	0	**0**	-		
	10	ATAAAGAACA	20	29	-1,65	-1,35	-0,30	0	0	**0**	-		
	8	AAAGAACA	25	24	-1,56	-1,27	-0,29	0	0	**0**	-		
	6	AGAACA	33	7	-1,07	-0,81	-0,26	0	0	**0**	-		

P240 RA-1 - DYS 389II	25	GCTGGTAGGCTCAGATAAAGAACAA	44	66	-12,78	-12,36	-0,42	300	300	**0**	-		
	24	CTGGTAGGCTCAGATAAAGAACAA	42	64	-11,29	-10,87	-0,42	298	296	**2**	-		
	23	TGGTAGGCTCAGATAAAGAACAA	39	62	-10,12	-9,70	-0,42	288	278	**10**	-		
	22	GGTAGGCTCAGATAAAGAACAA	41	62	-10,05	-9,63	-0,42	287	276	**11**	-		
	21	GTAGGCTCAGATAAAGAACAA	38	59	-8,47	-8,06	-0,41	198	153	**45**	-		
	20	TAGGCTCAGATAAAGAACAA	35	57	-7,53	-7,12	-0,41	95	60	**35**	-		
	19	AGGCTCAGATAAAGAACAA	37	58	-8,01	-7,59	-0,42	147	101	**46**	-		
	18	GGCTCAGATAAAGAACAA	39	56	-7,08	-6,66	-0,42	57	33	**24**	-		
	17	GCTCAGATAAAGAACAA	35	53	-5,77	-5,45	-0,32	9	6	**4**	-		
	16	CTCAGATAAAGAACAA	31	48	-4,29	-4,07	-0,22	1	1	**0**	-		
	15	TCAGATAAAGAACAA	27	45	-3,21	-3,00	-0,21	0	0	**0**	-		

probe	l	sequence	GC%	Tm NN	ΔG_M	ΔG_{MM}	$\Delta\Delta G_{M-MM}$	$con_M * 10^{-17}$ M	$con_{MM} * 10^{-17}$ M	$\Delta con_{M-MM} * 10^{-17}$ M	HSE result in %	SD	n
P244 FG - DYS 437	40	CTGGCATTCTAGCGCCCTGCCCAGCAGTGCAAC	65	85	-33,57	-32,51	-1,06	300	300	**0**	-		
	35	ATTCTAGCGCCCTGCCCAGCAGTGCAACAGGAC	63	83	-28,68	-27,62	-1,06	300	300	**0**	-		
	30	AGCGCCCTGCCCAGCAGTGCAACAGGACCG	70	84	-26,75	-25,70	-1,05	300	300	**0**	-		
	28	CGCCCTGCCCAGCAGTGCAACAGGACCG	71	83	-24,29	-23,23	-1,06	300	300	**0**	-		
	26	CCCTGCCCAGCAGTGCAACAGGACCG	69	80	-21,29	-20,23	-1,06	300	300	**0**	-		
	25	CCTGCCCAGCAGTGCAACAGGACCG	68	78	-20,00	-18,95	-1,05	300	300	**0**	-		
	23	TGCCCAGCAGTGCAACAGGACCG	65	75	-17,60	-16,54	-1,06	300	300	**0**	49	7	2
	22	GCCCAGCAGTGCAACAGGACCG	68	76	-17,54	-16,48	-1,06	300	300	**0**	-		
	21	CCCAGCAGTGCAACAGGACCG	67	73	-15,73	-14,67	-1,06	300	300	**0**	-		
	20	CCAGCAGTGCAACAGGACCG	65	71	-14,44	-13,39	-1,05	300	300	**0**	-		
	19	CAGCAGTGCAACAGGACCG	63	69	-13,19	-12,13	-1,06	300	299	**0**	-		
	18	AGCAGTGCAACAGGACCG	61	68	-12,60	-11,54	-1,06	300	299	**1**	-		
	17	GCAGTGCAACAGGACCG	65	67	-11,70	-10,65	-1,05	299	294	**4**	-		
	16	CAGTGCAACAGGACCG	63	64	-10,17	-9,21	-0,96	289	257	**32**	72	4	6
	15	AGTGCAACAGGACCG	60	62	-9,65	-8,70	-0,95	276	220	**56**	69	3	2
	14	GTGCAACAGGACCG	64	60	-8,86	-7,91	-0,95	234	136	**97**	71	5	3
	13	TGCAACAGGACCG	62	58	-7,97	-7,02	-0,95	143	53	**90**	73	6	2
	12	GCAACAGGACCG	67	57	-7,72	-6,77	-0,95	115	38	**77**	69	4	2
	11	CAACAGGACCG	64	51	-6,02	-5,06	-0,96	13	3	**10**	50	4	3
	10	AACAGGACCG	60	48	-5,50	-4,55	-0,95	6	2	**5**	-		
	9	ACAGGACCG	66	46	-5,32	-4,36	-0,96	5	1	**4**	-		
	8	CAGGACCG	75	40	-4,18	-3,51	-0,67	1	0	**1**	-		
	6	GGACCG	83	28	-3,08	-3,08	0,00	0	0	**0**	-		
P244 FG-1 - DYS 437	19	AGCAGTGCAACAGGACCGG	63	71	-13,86	-11,54	-2,32	300	299	**1**	-		
	18	GCAGTGCAACAGGACCGG	66	69	-12,96	-10,65	-2,31	300	294	**5**	-		
	17	CAGTGCAACAGGACCGG	64	66	-11,43	-9,21	-2,22	298	257	**41**	-		
	16	AGTGCAACAGGACCGG	62	65	-10,81	-8,70	-2,11	296	220	**75**	-		
	15	GTGCAACAGGACCGG	66	63	-10,02	-7,91	-2,11	286	136	**150**	78		1
	14	TGCAACAGGACCGG	64	61	-9,13	-7,02	-2,11	252	53	**199**	75	6	2
	13	GCAACAGGACCGG	69	61	-8,88	-6,77	-2,11	235	38	**197**	73		1
	12	CAACAGGACCGG	67	55	-7,17	-5,06	-2,11	64	3	**60**	-		
	11	AACAGGACCGG	64	53	-6,66	-4,55	-2,11	33	2	**32**	-		
P244 FA - DYS 437	23	GCCCAGCAGTGCAACAGGACCA	61	60	-16,46	-16,41	0,05	300	300	**0**	57	1	2
	21	CCCAGCAGTGCAACAGGACCA	62	71	-14,54	-14,59	0,05	300	300	**0**	-		
	20	CCAGCAGTGCAACAGGACCA	60	69	-13,26	-13,31	0,05	300	300	**0**	-		
	19	CAGCAGTGCAACAGGACCA	57	67	-12,00	-12,05	0,05	299	299	**0**	-		
	18	AGCAGTGCAACAGGACCA	55	66	-11,41	-11,46	0,05	298	298	**0**	-		
	17	GCAGTGCAACAGGACCA	58	64	-10,51	-10,56	0,05	293	294	**0**	-		
	16	CAGTGCAACAGGACCA	56	61	-9,03	-9,08	0,05	246	249	**-3**	49	4	2
	15	AGTGCAACAGGACCA	53	59	-8,52	-8,57	0,05	203	208	**-5**	-		
	14	GTGCAACAGGACCA	57	57	-7,72	-7,77	0,05	115	120	**-5**	54	6	2
	13	TGCAACAGGACCA	53	54	-6,83	-6,89	0,06	42	45	**-3**	-		
	22	CCCAGCAGTGCAACAGGACCAG	63	73	-15,78	-14,54	-1,24	300	300	**0**	-		
	21	CCAGCAGTGCAACAGGACCAG	62	71	-14,49	-13,26	-1,23	300	300	**0**	-		
	20	CAGCAGTGCAACAGGACCAG	60	69	-13,23	-12,00	-1,23	300	299	**1**	-		

	19	AGCAGTGCAACAGGACCAG	58	68	-12,64	-11,41	-1,23	300	298	**1**	-		
P244 FA-1 - DYS 437	18	GCAGTGCAACAGGACCAG	61	66	-11,75	-10,51	-1,24	299	293	**6**	-		
	17	CAGTGCAACAGGACCAG	58	63	-10,22	-9,08	-1,14	290	249	**40**	-		
	16	AGTGCAACAGGACCAG	56	62	-9,60	-8,57	-1,03	275	208	**67**	73	5	3
	15	GTGCAACAGGACCAG	60	60	-8,81	-7,77	-1,04	230	120	**109**	76	4	3
	14	TGCAACAGGACCAG	57	58	-7,92	-6,89	-1,03	137	45	**92**	-		
	22	ATTGTCCTGCAGCTCCATCCCC	59		-15,11	-14,36	-0,75	0	0	**0**	54	4	2
	18	TCCTGCAGCTCCATCCCC	66	69	-12,40	-11,64	-0,76	300	299	**1**	-		
	17	CCTGCAGCTCCATCCCC	70	69	-12,55	-11,80	-0,75	300	299	**1**	-		
P244 RC - DYS 437	16	CTGCAGCTCCATCCCC	68	65	-10,66	-10,01	-0,65	295	286	**9**	-		
	15	TGCAGCTCCATCCCC	66	62	-9,62	-8,96	-0,66	275	241	**34**	62		1
	14	GCAGCTCCATCCCC	71	63	-9,65	-9,00	-0,65	276	244	**32**	68		1
	13	CAGCTCCATCCCC	69	58	-7,95	-7,29	-0,66	141	73	**67**	63		1
	12	AGCTCCATCCCC	67	56	-7,43	-6,78	-0,65	85	39	**47**	-		
	19	TCCTGCAGCTCCATCCCCG	68	72	-14,00	-11,64	-2,36	300	299	**1**	-		
	18	CCTGCAGCTCCATCCCCG	72	72	-14,15	-11,80	-2,35	300	299	**1**	-		
	17	CTGCAGCTCCATCCCCG	71	68	-12,26	-10,01	-2,25	300	286	**14**	-		
P244 RC-1 - DYS 437	16	TGCAGCTCCATCCCCG	69	66	-11,12	-8,96	-2,16	297	241	**56**	-		
	15	GCAGCTCCATCCCCG	73	66	-11,15	-9,00	-2,15	297	244	**53**	-		
	14	CAGCTCCATCCCCG	71	62	-9,45	-7,29	-2,16	269	73	**195**	71		1
	13	AGCTCCATCCCCG	69	61	-8,93	-6,78	-2,15	239	39	**200**	69	4	3
	12	GCTCCATCCCCG	75	58	-8,14	-5,98	-2,16	162	13	**149**	-		
	11	CTCCATCCCCG	72	53	-6,70	-4,55	-2,15	35	1	**33**	-		
	22	ATTGTCCTGCAGCTCCATCCCT	55	72	-14,93	-14,24	-0,69	300	300	**0**	52	0	2
	21	TTGTCCTGCAGCTCCATCCCT	57	70	-13,78	-13,09	-0,69	300	300	**0**			
	20	TGTCCTGCAGCTCCATCCCT	60	70	-13,90	-13,21	-0,69	300	300	**0**			
	19	GTCCTGCAGCTCCATCCCT	63	71	-13,47	-12,79	-0,68	300	300	**0**			
	18	TCCTGCAGCTCCATCCCT	61	69	-12,21	-11,53	-0,68	299	299	**1**	-		
	17	CCTGCAGCTCCATCCCT	64	69	-12,37	-11,68	-0,69	300	299	**1**	-		
P244 RT - DYS 437	16	CTGCAGCTCCATCCCT	62	65	-10,47	-9,79	-0,68	293	281	**12**	55	9	5
	15	TGCAGCTCCATCCCT	60	62	-9,43	-8,74	-0,69	268	224	**44**	-		
	14	GCAGCTCCATCCCT	64	62	-9,47	-8,78	-0,69	270	227	**43**	-		
	13	CAGCTCCATCCCT	61	57	-7,76	-7,07	-0,69	119	56	**63**	62	8	4
	12	AGCTCCATCCCT	58	55	-7,25	-6,56	-0,69	70	29	**41**	-		
	11	GCTCCATCCCT	63	52	-6,45	-5,77	-0,68	25	9	**16**	54		1
	10	CTCCATCCCT	60	45	-5,02	-4,33	-0,69	3	1	**2**	-		
	9	TCCATCCCT	55	39	-3,95	-3,26	0,00	1	0	**0**	-		
	19	TCCTGCAGCTCCATCCCTG	63	69	-12,79	-11,67	-1,12	300	299	**1**	-		
	18	CCTGCAGCTCCATCCCTG	66	70	-12,94	-11,83	-1,11	300	299	**1**	-		
	17	CTGCAGCTCCATCCCTG	65	65	-11,05	-9,93	-1,12	297	284	**13**	-		
P244 RT-1 - DYS 437	16	TGCAGCTCCATCCCTG	63	63	-9,90	-8,89	-1,01	283	236	**47**	-		
	15	GCAGCTCCATCCCTG	67	63	-9,94	-8,93	-1,01	284	239	**45**	-		
	14	CAGCTCCATCCCTG	57	59	-8,23	-7,22	-1,01	172	68	**105**	66	3	4
	13	AGCTCCATCCCTG	62	57	-7,72	-6,71	-1,01	115	35	**80**	-		
	12	GCTCCATCCCTG	67	54	-6,93	-5,91	-1,02	47	11	**36**	-		
	11	CTCCATCCCTG	64	48	-5,49	-4,48	-1,01	6	1	**5**	-		

probe	l	sequence	GC%	Tm NN	ΔG_M	ΔG_{MM}	$\Delta\Delta G_{M\text{-}MM}$	$con_M*10^{-17}M$	$con_{MM}*10^{-17}M$	$\Delta con_{M\text{-}MM}*10^{-17}M$	HSE result in %	SD	n
1304202 FG - DYS635	25	TATCTAAGGAACATTACTCAAGAGG	36	62	-10,38	-8,99	-1,39	292	243	**49**	**-**		
	24	ATCTAAGGAACATTACTCAAGAGG	38	63	-10,79	-9,40	-1,39	296	267	**29**	-		
	23	TCTAAGGAACATTACTCAAGAGG	39	61	-9,64	-8,25	-1,39	276	175	**101**	-		
	22	CTAAGGAACATTACTCAAGAGG	41	62	-10,21	-8,82	-1,39	289	230	**59**	-		
	21	TAAGGAACATTACTCAAGAGG	38	59	-8,59	-7,20	-1,39	210	66	**144**	**75**	9	2
	20	AAGGAACATTACTCAAGAGG	40	61	-9,19	-7,80	-1,39	256	124	**132**	-		
	19	AGGAACATTACTCAAGAGG	42	60	-8,86	-7,47	-1,39	234	90	**144**	-		
	18	GGAACATTACTCAAGAGG	44	58	-7,89	-6,50	-1,39	134	27	**107**	-		
	17	GAACATTACTCAAGAGG	41	55	-6,58	-5,19	-1,39	30	4	**26**	-		
	16	AACATTACTCAAGAGG	38	53	-6,26	-4,97	-1,29	19	3	**16**	-		
	15	ACATTACTCAAGAGG	40	52	-6,00	-4,71	-1,29	13	2	**11**	**-**		

probe	l	sequence	GC%	Tm NN	ΔG_M	ΔG_{MM}	$\Delta\Delta G_{M\text{-}MM}$	$con_M*10^{-17}M$	$con_{MM}*10^{-17}M$	$\Delta con_{M\text{-}MM}*10^{-17}M$	HSE result in %	SD	n
1304202 FG-1 - DYS635	26	TATCTAAGGAACATTACTCAAGAGGC	39	64	-11,89	-9,12	-2,77	299	252	**47**	**-**		
	25	ATCTAAGGAACATTACTCAAGAGGC	40	65	-12,29	-9,53	-2,76	300	272	**27**	-		
	24	TCTAAGGAACATTACTCAAGAGGC	42	64	-11,15	-8,38	-2,77	297	189	**109**	-		
	23	CTAAGGAACATTACTCAAGAGGC	44	65	-11,71	-8,95	-2,76	299	240	**58**	-		
	22	TAAGGAACATTACTCAAGAGGC	41	62	-10,10	-7,33	-2,77	288	77	**211**	-		
	21	AAGGAACATTACTCAAGAGGC	43	63	-10,70	-7,93	-2,77	295	138	**157**	-		
	20	AGGAACATTACTCAAGAGGC	45	63	-10,37	-7,61	-2,76	292	103	**188**	-		
	19	GGAACATTACTCAAGAGGC	47	61	-9,40	-6,63	-2,77	267	32	**235**	**81**	11	2
	18	GAACATTACTCAAGAGGC	44	58	-8,09	-5,32	-2,77	157	5	**152**	-		
	17	AACATTACTCAAGAGGC	41	57	-7,77	-5,00	-2,77	120	3	**118**	-		
	16	ACATTACTCAAGAGGC	44	56	-7,41	-4,74	-2,67	84	2	**82**	**-**		

probe	l	sequence	GC%	Tm NN	ΔG_M	ΔG_{MM}	$\Delta\Delta G_{M\text{-}MM}$	$con_M*10^{-17}M$	$con_{MM}*10^{-17}M$	$\Delta con_{M\text{-}MM}*10^{-17}M$	HSE result in %	SD	n
1304202 FA - DYS635	40	ATATTTGAAGAGGTTTATCTAAGGAACATTACTCAAGAGA	30	68	-16,87	-17,29	0,42	300	300	**0**	**-**		
	35	TGAAGAGGTTTATCTAAGGAACATTACTCAAGAGA	34	67	-15,22	-15,64	0,42	300	300	**0**	-		
	30	AGGTTTATCTAAGGAACATTACTCAAGAGA	33	65	-12,45	-12,86	0,41	300	300	**0**	-		
	28	GTTTATCTAAGGAACATTACTCAAGAGA	32	61	-10,17	-10,59	0,42	289	294	**-5**	-		
	26	TTATCTAAGGAACATTACTCAAGAGA	31	60	-8,94	-9,35	0,41	240	264	**-25**	-		
	25	TATCTAAGGAACATTACTCAAGAGA	32	59	-8,57	-8,99	0,42	208	243	**-35**	-		
	24	ATCTAAGGAACATTACTCAAGAGA	33	60	-8,97	-9,39	0,42	242	266	**-24**	-		
	23	TCTAAGGAACATTACTCAAGAGA	35	58	-7,82	-8,24	0,42	126	173	**-47**	-		
	22	CTAAGGAACATTACTCAAGAGA	36	59	-8,39	-8,81	0,42	190	230	**-40**	-		
	21	TAAGGAACATTACTCAAGAGA	33	55	-6,78	-7,20	0,42	39	66	**-27**	**73**	7	2
	20	AAGGAACATTACTCAAGAGA	35	57	-7,38	-7,80	0,42	81	124	**-43**	**72**	4	2
	19	AGGAACATTACTCAAGAGA	37	56	-7,05	-7,47	0,42	55	90	**-34**	**77**	11	4
	18	GGAACATTACTCAAGAGA	39	53	-6,07	-6,49	0,42	14	26	**-12**	**70**	13	3
	17	GAACATTACTCAAGAGA	35	49	-4,77	-5,19	0,42	2	4	**-2**	-		
	16	AACATTACTCAAGAGA	31	48	-4,55	-4,97	0,42	2	3	**-1**	-		
	15	ACATTACTCAAGAGA	33	46	-4,28	-4,70	0,42	1	2	**-1**	-		
	14	CATTACTCAAGAGA	36	43	-3,15	-3,57	0,42	0	0	**0**	-		
	13	ATTACTCAAGAGA	31	39	-2,79	-3,21	0,42	0	0	**0**	-		
	12	TTACTCAAGAGA	33	29	-1,92	-2,34	0,42	0	0	**0**	-		
	11	TACTCAAGAGA	36	33	-2,01	-2,43	0,42	0	0	**0**	-		
	10	ACTCAAGAGA	40	32	-2,51	-2,93	0,42	0	0	**0**	-		

	8	TCAAGAGA	38	16	-0,51	-0,93	0,42	0	0	**0**	**-**		

	26	TATCTAAGGAACATTACTCAAGAGAC	34	62	-10,54	-8,99	-1,55	294	243	**50**	**-**		
	25	ATCTAAGGAACATTACTCAAGAGAC	36	63	-10,95	-9,39	-1,56	296	266	**30**	-		
	24	TCTAAGGAACATTACTCAAGAGAC	38	61	-9,80	-8,24	-1,56	281	173	**107**	-		
	23	CTAAGGAACATTACTCAAGAGAC	39	62	-10,37	-8,81	-1,56	292	230	**62**	-		
1304202 FA-1 - DYS635	22	TAAGGAACATTACTCAAGAGAC	36	60	-8,75	-7,20	-1,55	225	66	**159**	**76**	8	2
	21	AAGGAACATTACTCAAGAGAC	38	61	-9,35	-7,80	-1,55	264	124	**141**	-		
	20	AGGAACATTACTCAAGAGAC	40	60	-9,02	-7,47	-1,55	245	90	**156**	**73**	10	2
	19	GGAACATTACTCAAGAGAC	42	58	-8,05	-6,49	-1,56	152	26	**126**	-		
	18	GAACATTACTCAAGAGAC	39	55	-6,74	-5,19	-1,55	37	4	**33**	-		
	17	AACATTACTCAAGAGAC	35	54	-6,42	-4,97	-1,45	24	3	**21**	-		
	16	ACATTACTCAAGAGAC	38	53	-6,06	-4,70	-1,36	14	2	**12**	**-**		

	40	GAATTTCATTAGCTGAATTAGTTTTATTTATGGAGGAAGC	30	69	-17,97	-16,80	-1,17	300	300	**0**	**-**		
	35	TCATTAGCTGAATTAGTTTTATTTATGGAGGAAGC	31	68	-15,80	-14,64	-1,16	300	300	**0**	-		
	30	AGCTGAATTAGTTTTATTTATGGAGGAAGC	33	67	-14,08	-12,91	-1,17	300	300	**0**	-		
	28	CTGAATTAGTTTTATTTATGGAGGAAGC	32	64	-11,61	-10,44	-1,17	299	292	**6**	-		
	26	GAATTAGTTTTATTTATGGAGGAAGC	31	62	-10,37	-9,20	-1,17	292	257	**35**	-		
	25	AATTAGTTTTATTTATGGAGGAAGC	28	61	-9,73	-8,56	-1,17	279	207	**72**	-		
	24	ATTAGTTTTATTTATGGAGGAAGC	29	60	-9,36	-8,20	-1,16	265	169	**96**	-		
	23	TTAGTTTTATTTATGGAGGAAGC	30	58	-8,10	-6,94	-1,16	158	48	**110**	-		
	22	TAGTTTTATTTATGGAGGAAGC	32	59	-8,30	-7,13	-1,17	180	61	**119**	-		
	21	AGTTTTATTTATGGAGGAAGC	33	59	-8,70	-7,53	-1,17	220	95	**125**	**70**		1
1304202 RC - DYS635	20	GTTTTATTTATGGAGGAAGC	35	58	-7,77	-6,60	-1,17	120	31	**90**	-		
	19	TTTTATTTATGGAGGAAGC	32	55	-6,78	-5,61	-1,17	39	7	**32**	**62**	15	3
	18	TTTATTTATGGAGGAAGC	33	54	-6,49	-5,32	-1,17	26	5	**21**	**61**	6	2
	17	TTATTTATGGAGGAAGC	35	53	-6,12	-4,95	-1,17	16	3	**13**	-		
	16	TATTTATGGAGGAAGC	38	52	-5,75	-4,68	-1,07	9	2	**7**	-		
	15	ATTTATGGAGGAAGC	40	53	-6,25	-5,19	-1,06	19	4	**15**	-		
	14	TTTATGGAGGAAGC	43	50	-5,21	-4,14	-1,07	4	1	**3**	-		
	13	TTATGGAGGAAGC	46	50	-5,43	-4,36	-1,07	6	1	**5**	-		
	12	TATGGAGGAAGC	50	48	-5,16	-4,10	-1,06	4	1	**3**	-		
	11	ATGGAGGAAGC	55	49	-5,67	-4,60	-1,07	8	2	**6**	-		
	10	TGGAGGAAGC	60	45	-4,62	-3,55	-1,07	2	0	**1**	-		
	8	GAGGAAGC	63	35	-3,31	-2,24	-1,07	0	0	**0**	-		
	6	GGAAGC	67	24	-2,22	-1,15	-1,07	0	0	**0**	**-**		

	40	GAATTTCATTAGCTGAATTAGTTTTATTTATGGAGGAAGCC	32	70	-19,27	-16,80	-2,47	300	300	**0**	**-**		
	35	TCATTAGCTGAATTAGTTTTATTTATGGAGGAAGCC	33	69	-17,10	-14,64	-2,46	300	300	**0**	-		
1304202 RC-1 - DYS635	30	AGCTGAATTAGTTTTATTTATGGAGGAAGCC	35	68	-15,37	-12,91	-2,46	300	300	**0**	-		
	28	CTGAATTAGTTTTATTTATGGAGGAAGCC	34	65	-12,90	-10,44	-2,46	300	292	**7**	-		
	26	AATTAGTTTTATTTATGGAGGAAGCC	31	63	-11,02	-8,56	-2,46	297	207	**90**	-		
	25	ATTAGTTTTATTTATGGAGGAAGCC	32	63	-10,66	-8,20	-2,46	295	169	**126**	-		
	24	TTAGTTTTATTTATGGAGGAAGCC	33	61	-9,40	-6,94	-2,46	267	48	**219**	**74**	17	2
	23	TAGTTTTATTTATGGAGGAAGCC	35	61	-9,59	-7,13	-2,46	274	61	**214**	-		
	22	AGTTTTATTTATGGAGGAAGCC	36	62	-9,99	-7,53	-2,46	285	95	**190**	-		
	21	GTTTTATTTATGGAGGAAGCC	38	60	-9,06	-6,60	-2,46	248	31	**217**	**81**	8	2

	20	TTTTATTTATGGAGGAAGCC	35	58	-8,07	-5,61	-2,46	154	7	**147**	**77**	1
	19	TTTATTTATGGAGGAAGCC	37	57	-7,78	-5,32	-2,46	122	5	**117**	**77**	1
	18	TTATTTATGGAGGAAGCC	39	57	-7,41	-4,95	-2,46	84	3	**81**	-	
	17	TATTTATGGAGGAAGCC	41	56	-7,04	-4,68	-2,36	54	2	**53**	-	
	16	ATTTATGGAGGAAGCC	44	57	-7,45	-5,19	-2,26	88	4	**84**	-	
	15	ATTTATGGAGGAAGCC	44	57	-7,45	-5,19	-2,26	88	4	**84**	-	
1304202 RC-1 - DYS635	14	TTTATGGAGGAAGCC	47	54	-6,40	-4,14	-2,26	23	1	**22**	-	
	13	TTATGGAGGAAGCC	50	54	-6,62	-4,36	-2,26	31	1	**30**	-	
	12	TATGGAGGAAGCC	54	53	-6,36	-4,10	-2,26	22	1	**21**	-	
	11	ATGGAGGAAGCC	58	54	-6,86	-4,60	-2,26	43	2	**42**	-	
	10	TGGAGGAAGCC	64	50	-5,81	-3,55	-2,26	10	0	**10**	-	
	8	GAGGAAGCC	67	43	-4,50	-2,24	-2,26	1	0	**1**	**-**	
	6	GGAAGCC	71	34	-3,41	-1,70	-1,71	0	0	**0**	**-**	

probe	l	sequence	GC%	Tm NN	ΔG_M	ΔG_{MM}	$\Delta\Delta G_{M-MM}$	$con_M * 10^{-17} M$	$con_{MM} * 10^{-17} M$	$\Delta con_{M-MM} * 10^{-17} M$	HSE result in %	SD	n
Tat FT - DYS 438	**23**	TGAGTGTAGACTTGTGAATTCAT	34	61,6	-9,95	-9,38	-0,57	285	266	**19**	**-**		
	**22**	GAGTGTAGACTTGTGAATTCAT	36	61,6	-9,89	-9,32	-0,57	283	263	**20**	-		
	**21**	AGTGTAGACTTGTGAATTCAT	33	60,6	-9,25	-8,68	-0,57	259	218	**41**	-		
	**20**	GTGTAGACTTGTGAATTCAT	35	58,5	-8,28	-7,71	-0,57	178	114	**64**	**77**	4	3
	**19**	TGTAGACTTGTGAATTCAT	31	56,2	-7,29	-6,72	-0,57	73	36	**37**	-		
	**18**	GTAGACTTGTGAATTCAT	33	55,5	-6,94	-6,37	-0,57	48	22	**26**	-		
	**17**	TAGACTTGTGAATTCAT	29	51,8	-5,68	-5,11	-0,57	8	3	**5**	-		
	**16**	AGACTTGTGAATTCAT	31	52,9	-6,15	-5,69	-0,46	16	8	**8**	**-**		
Tat FC - DYS 438	**22**	GAGTGTAGACTTGTGAATTCAC	41	61,7	-10,01	-9,49	-0,52	286	270	**15**	**-**		
	**21**	AGTGTAGACTTGTGAATTCAC	38	60,8	-9,37	-8,85	-0,52	263	230	**34**	-		
	**20**	GTGTAGACTTGTGAATTCAC	40	58,8	-8,40	-7,87	-0,53	191	132	**59**	**85**	7	2
	**19**	TGTAGACTTGTGAATTCAC	37	56,6	-7,41	-6,89	-0,52	84	45	**39**	-		
	**18**	GTAGACTTGTGAATTCAC	38	55,8	-7,06	-6,54	-0,52	56	28	**28**	**87**	1	3
	**17**	TAGACTTGTGAATTCAC	35	52,3	-5,80	-5,27	-0,53	10	4	**5**	-		
	**16**	AGACTTGTGAATTCAC	38	53,4	-6,27	-5,85	-0,42	19	11	**9**	-		
	**15**	GACTTGTGAATTCAC	40	51,2	-5,44	-5,02	-0,42	6	3	**3**	**-**		

probe	l	sequence	GC%	Tm NN	ΔG_M	ΔG_{MM}	$\Delta\Delta G_{M-MM}$	$con_M * 10^{-17}M$	$con_{MM} * 10^{-17}M$	$\Delta con_{M-MM} * 10^{-17}M$	HSE result in %	SD	n
	**23**	AGGCTGTGCTATTGATGAAAATG	39	67,1	-13,01	-11,81	-1,20	300	299	**1**	**67**		1
	**21**	GCTGTGCTATTGATGAAAATG	41	65,5	-12,07	-10,88	-1,19	299	296	**3**	-		
Seq.E	**20**	CTGTGCTATTGATGAAAATG	38	63,3	-10,72	-9,53	-1,19	295	272	**23**	**78**		1
2071 RG	**19**	TGTGCTATTGATGAAAATG	35	60,4	-9,19	-8,00	-1,19	256	146	**110**	-		
-	**18**	GTGCTATTGATGAAAATG	32	57,9	-7,98	-6,79	-1,19	144	39	**105**	-		
DYS 437	**17**	TGCTATTGATGAAAATG	33	57,6	-7,87	-6,68	-1,19	132	34	**98**	-		
	**16**	GCTATTGATGAAAATG	29	54,3	-6,57	-5,38	-1,19	29	5	**24**	-		
	**15**	CTATTGATGAAAATG	31	53,4	-6,18	-5,09	-1,09	17	3	**14**	**-**		
	**23**	AGGCTGTGCTATTGATGAAAATA	35	65,2	-11,90	-11,77	-0,13	299	299	**0**	**77**		1
	**22**	GGCTGTGCTATTGATGAAAATA	36	63,6	-10,97	-10,83	-0,14	296	296	**1**	-		
Seq.E	**21**	GCTGTGCTATTGATGAAAATA	33	61,2	-9,62	-9,49	-0,13	275	270	**5**	-		
2071 RA	**20**	CTGTGCTATTGATGAAAATA	30	58,1	-8,09	-7,96	-0,13	157	142	**15**	**91**		1
-	**19**	TGTGCTATTGATGAAAATA	26	55,5	-6,88	-6,74	-0,14	44	37	**7**	-		
DYS 437	**18**	GTGCTATTGATGAAAATA	28	55,1	-6,77	-6,64	-0,13	38	32	**6**	-		
	**17**	TGCTATTGATGAAAATA	24	51,4	-5,47	-5,34	-0,13	6	5	**1**	-		
	**16**	GCTATTGATGAAAATA	25	50,5	-5,08	-5,04	-0,04	3	3	**0**	**-**		
	**20**	GGTCTTCCTCTGTTCCTCAG	55	66	-11,83	-11,12	-0,71	266	219	**48**	**49**	1	2
	**19**	GTCTTCCTCTGTTCCTCAG	53	63,5	-10,53	-9,82	-0,71	262	210	**52**	-		
Seq.F	**18**	TCTTCCTCTGTTCCTCAG	50	61,7	-9,55	-8,84	-0,71	245	181	**64**	-		
4204 FG	**17**	CTTCCTCTGTTCCTCAG	53	62	-9,66	-8,95	-0,71	249	186	**62**	**67**	2	4
-	**16**	TTCCTCTGTTCCTCAG	50	57,5	-7,84	-7,23	-0,61	122	64	**58**	-		
DYS 437	**15**	TCCTCTGTTCCTCAG	53	57,6	-7,89	-7,28	-0,61	127	67	**59**	-		
	**14**	CCTCTGTTCCTCAG	57	58	-8,02	-7,42	-0,60	140	78	**62**	-		
	**13**	CTCTGTTCCTCAG	54	52,3	-6,19	-5,58	-0,61	17	7	**10**	**-**		
	**20**	GGTCTTCCTCTGTTCCTCAC	55	65,9	-11,79	-10,96	-0,83	289	264	**25**	**88**	4	2
	**19**	GTCTTCCTCTGTTCCTCAC	53	63,4	-10,49	-9,66	-0,83	283	249	**35**	-		
Seq.F	**18**	TCTTCCTCTGTTCCTCAC	50	61,6	-9,51	-8,68	-0,83	263	200	**63**	-		
4204 FC	**17**	CTTCCTCTGTTCCTCAC	53	61,9	-9,62	-8,79	-0,83	267	208	**58**	**98**	1	2
-	**16**	TTCCTCTGTTCCTCAC	50	57,4	-7,80	-7,07	-0,73	122	55	**67**	-		
DYS 437	**15**	TCCTCTGTTCCTCAC	53	57,5	-7,85	-7,12	-0,73	127	59	**69**	-		
	**14**	CCTCTGTTCCTCAC	57	57,9	-7,99	-7,26	-0,73	142	69	**74**	-		
	**13**	CTCTGTTCCTCAC	54	52,2	-6,16	-5,43	-0,73	16	6	**11**	**-**		
