# Supplementary material for: New Prediction Model for Probe Specificity in an Allele-Specific Extension Reaction for Haplotype-Specific Extraction (HSE) of Y Chromosome Mixtures
Source: PLoS One. 2012 Sep 25;7(9):e45955. doi: 10.1371/journal.pone.0045955 (PMC3457965; doi:10.1371/journal.pone.0045955)
Supplement: File S4 — Figure. (DOC) [file pone.0045955.s004.doc]

**Supplement 4**


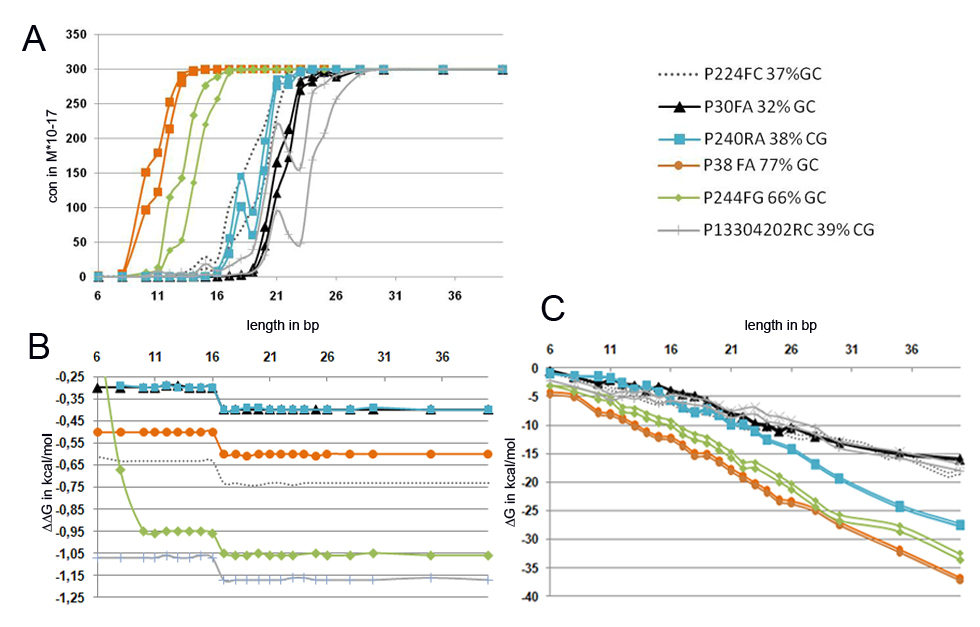
Comparison of probes with different GC contents. GC contents has been calculated as mean values for all simulated probe-length of one probe-set. (A) Concentration of match and mismatch increase with probe-length and GC contents. (B) Difference of ΔG between match and mismatch stay constant for increasing probe-length and GC contents. The observed shift in the change in free energy at a probe length of 16 nucleotides for each of the probes is attributed to a polymer salt effect in DNA duplexes that was previously reported in the literature (21). (C) ΔG decrease with increasing probe-length and CG contents.
